# Retinoic acid degradation shapes zonal development of vestibular organs and sensitivity to transient linear accelerations

**DOI:** 10.1038/s41467-019-13710-4

**Published:** 2020-01-02

**Authors:** Kazuya Ono, James Keller, Omar López Ramírez, Antonia González Garrido, Omid A. Zobeiri, Hui Ho Vanessa Chang, Sarath Vijayakumar, Andrianna Ayiotis, Gregg Duester, Charles C. Della Santina, Sherri M. Jones, Kathleen E. Cullen, Ruth Anne Eatock, Doris K. Wu

**Affiliations:** 1 National Institute on Deafness and Other Communication Disorders, National Institutes of Health, Bethesda, MD 20892 USA; 2 Department of Neurobiology, University of Chicago, Chicago, IL 60637 USA; 3 Department of Physiology McGill University, Montreal, QC Canada H3G 1Y6; 4 Department of Special Education and Communication Disorders, 301 Barkley Memorial Center, University of Nebraska-Lincoln, Lincoln, NE 68583-0738 USA; 5 Department of Biomedical Engineering, Johns Hopkins University School of Medicine, Baltimore, MD 21205 USA; 6 Neuroscience and Aging Research Center, Stanford Burnham Prebys Medical Discovery Institutes, Stanford, CA 92037 USA; 7 Department of Otolaryngology—Head and Neck Surgery, Johns Hopkins University School of Medicine, Baltimore, MD 21205 USA; 8 Present Address: Qiagen Sciences Inc., Germantown, MD 20874 USA

## Abstract

Each vestibular sensory epithelium in the inner ear is divided morphologically and physiologically into two zones, called the striola and extrastriola in otolith organ maculae, and the central and peripheral zones in semicircular canal cristae. We found that formation of striolar/central zones during embryogenesis requires Cytochrome P450 26b1 (Cyp26b1)-mediated degradation of retinoic acid (RA). In *Cyp26b1* conditional knockout mice, formation of striolar/central zones is compromised, such that they resemble extrastriolar/peripheral zones in multiple features. Mutants have deficient vestibular evoked potential (VsEP) responses to jerk stimuli, head tremor and deficits in balance beam tests that are consistent with abnormal vestibular input, but normal vestibulo-ocular reflexes and apparently normal motor performance during swimming. Thus, degradation of RA during embryogenesis is required for formation of highly specialized regions of the vestibular sensory epithelia with specific functions in detecting head motions.

## Abstract (web summary)

The coding of sensory inputs at the level of vestibular sensory organs is not well understood. In this study, the authors demonstrate that the formation of striolar/central zones during embryogenesis requires Cytochrome P450 26b1 (Cyp26b1)-mediated degradation of retinoic acid and show that *Cyp26b1* cKO mice have abnormal vestibular evoked potentials and balance beam performance, but normal vestibular-ocular reflexes.

## Introduction

Sense of balance and heading is mediated by integration of vestibular, visual, and proprioceptive inputs. While unilateral vestibular deficits can largely be compensated by sensorimotor reorganization, bilateral loss of vestibular inner ear function, such as caused by aminoglycoside ototoxicity, cannot be fully compensated^1^. As a consequence, patients with chronic bilateral vestibulopathy are disabled by imbalance and oscillopsia. Understanding how vestibular inputs are encoded by vestibular organs to maintain gaze and head stability is important from basic science and therapeutic perspectives.

**Fig. 1:**
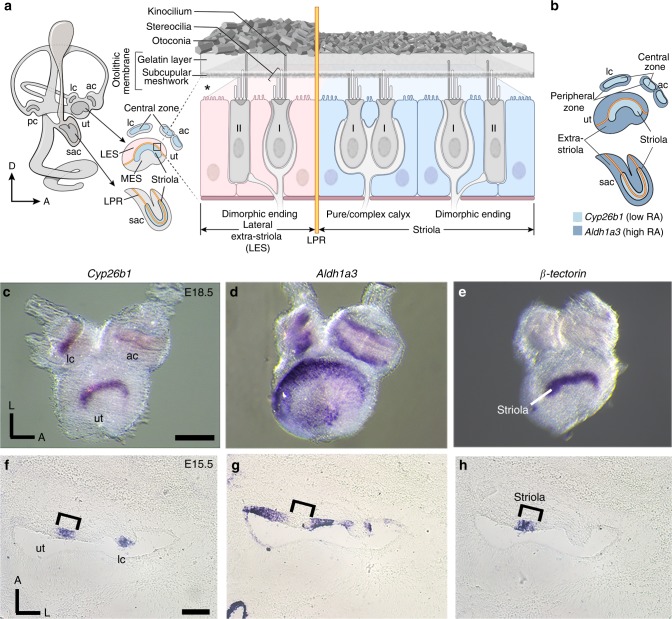
Complementary expression patterns of *Cyp26b1* and *Aldh1a3*. **a** Schematic illustration of the inner ear and sectional view of the utricle (ut) across the striola and lateral extrastriola region (LES). Pear-shaped type I HCs are innervated by calyces and cylindrical-shaped type II HCs are innervated by bouton type endings. Pure/complex calyces are exclusively present in the striolar/central zone, whereas dimorphic nerve endings are found across the entire organ. Otoconia are smaller in size and less abundant in striola than extrastriola of the utricle. Asterisk indicates that the relationship between hair bundles and the otoconial membrane is not clear, but evidence suggests that hair bundles of striolar HCs are less firmly embedded in the otolithic membrane than their counterparts in the extrastriolar region^9,84^. Yellow line represents the line of polarity reversal (LPR), which separates each macula into two regions with opposite hair bundle orientations: 1) striola and MES (medial extrastriola): 2) LES. The striola of maculae and the central zone of anterior (ac) and lateral cristae (lc) are in blue color. The LPR is at the lateral edge of the striola in the ut but it bisects the striola in the saccule (sac). **b** Schematic summary of the expession pattern of *Cyp26b1* (light blue) and *Aldh1a3* (dark blue) described in **c**–**h**. **c**–**e** Whole-mount in situ hybridization analysis of *Cyp26b1*, *Aldh1a3*, and β-*tectorin* transcripts at E18.5 mouse ut, ac, and lc. Expression of *Cyp26b1* (**c**) is restricted to the central zone of the two cristae and striola of the ut that is β-*tectorin* positive **e**, whereas *Aldh1a3* (**d**) is predominantly expressed in the peripheral regions. **f**–**h** Adjacent tissue sections at the levels of ut and lc at E15.5. **f** *Cyp26b1* expression is concentrated in the supporting cell (SC) layer of the central zone of the lc and striola of the ut, comparable to the β-tectorin domain (**h**, bracket). **g** *Aldh1a3* expression is largely complementary to *Cyp26b1* (**f**) in each organ. Scale bars: 200 μm. RA, retinoic acid; D, dorsal; A, anterior; L, lateral; pc, posterior crista. **a**, **b** Drawn by NIH Medical Arts.

Vestibular sensory epithelia comprise the maculae of the utricle and saccule, which detect linear acceleration, and the three canal cristae, which detect angular acceleration. Each sensory epithelium contains type I and type II mechanosensitive hair cells (HCs), which are surrounded by supporting cells (SCs) and innervated by afferent neurons of the vestibular ganglion (Fig. 1a). Type I and II HCs are contacted by different afferent synaptic terminals: large calyceal endings on type I HCs and small bouton endings on type II HCs. The mechanosensitive stereociliary (hair) bundles of HCs couple to an otolithic membrane in the maculae and to a cupula in the cristae. Head accelerations deflect these accessory structures and the coupled hair bundles, modulating mechanotransduction channels in the bundles and ultimately changing the firing rate of afferent neurons^2^.

Each vestibular organ has near its center a conserved, specialized region called the striola in the maculae and the central zone in the cristae^3–5^. Striolar/central zones may have evolved in land-based vertebrates as an adaptation to changes required for locomotion, including large independent head movements with high-frequency components^6,7^. Striolas and central zones differ from extrastriolas and peripheral zones in many features, including hair bundle morphology, ion channel expression, and otoconia size^8–10^. Additionally, afferents form complex calyces around multiple type I HCs in greater proportion in striolar/central zones (Fig. 1a)^4,5,11,12^. Such differences give rise to afferent nerve populations with very different spontaneous and evoked physiological responses. Striolar/central zone afferents have more irregular spike timing and are more sensitive to higher-frequency head motion than extrastriolar/peripheral afferents^6,13,14^. Higher densities of low-voltage-activated K (K_LV_) channels are expressed in striolar/central zone afferents, making them less excitable—less likely to fire in response to small currents—which contributes to their irregular firing patterns^15^. By virtue of their different regularities, afferents from the two zones encode head motion into spike trains by different strategies: temporal pattern of spikes for the striolar/central zones vs. spike rate for extrastriolar/peripheral zones^16^, which are optimal for different kinds of sensory information. The formation of these regional specializations and their significance in mediating vestibular functions such as the vestibulo-ocular and vestibulo-spinal reflexes (VOR and VSR) are not known. Nevertheless, several lines of indirect evidence implicate irregular afferents, which innervate the striola, in generating the vestibular-evoked potential (VsEP)^2,17–19^, a potential recorded in vivo that reflects activation of macular afferents by linear head motions^17,20,21^.

Here, we show that formation of the striolar/central zones of vestibular organs requires degradation of retinoic acid (RA) by the zone-specific expression of *Cyp26b1*, a gene encoding a RA degradation enzyme. RA, an important morphogen during embryogenesis^22,23^, is the bioactive form of vitamin A (retinol), which binds to RA receptors in the nucleus to regulate transcription. The availability of RA during embryogenesis is controlled by restricted expression of RA-synthesizing enzymes such as class 1A aldehyde dehydrogenases (Aldh1a), as well as degradation enzymes such as Cyp26s^22^. The complementary expression patterns of these enzymes during embryogenesis are important in patterning many tissues including the anterior–posterior axis of the inner ear^24–27^. We show that *Cyp26b1* conditional knockout (cKO) mice exhibit a severe reduction of striolar/central zones as manifested by a number of morphological, molecular, and physiological properties. These mice have normal horizontal angular vestibular-ocular reflexes (aVOR), driven by horizontal canal cristae, and normal responses to off-vertical axis rotation (OVAR), driven by the macular organs, but they lack VsEP and have head tremor and deficits during balance beam tests. These results suggest that striolas and central zones are not essential for mediating VORs, but are important for responding to changes in linear acceleration and are likely important for controlling head stability and performing challenging vestibulomotor activities.

## Results

### Expression of *Cyp26b1* and *Aldh1a3* in the vestibular organs

We observed complementary expression patterns of transcripts for the RA-degrading enzyme Cyp26b1 and the RA-synthesizing enzyme Aldh1a3 in developing vestibular organs that were not described previously. During embryogenesis, the sensory epithelia of the utricle (Fig. 1b–d), saccule (Fig. 1b, Supplementary Fig. 1), and cristae (Fig. 1b–d) show *Cyp26b1* expression in the center surrounded by *Aldh1a3* expression in the periphery. The *Cyp26b1* expression domain in the maculae appears comparable to that of β-tectorin, a striolar SC marker (Fig. 1c, e)^28^. Adjacent cryo-sections processed for in situ hybridization confirmed the complementary relationships among *Aldh1a3*, *Cyp26b1*, and β-tectorin in both maculae and the lateral crista (Fig.1f–h, Supplementary Fig. 1). *Cyp26b1* hybridization signals appear to be concentrated basally in striolar SCs, but *Aldh1a3* distribution is broad in the extrastriola/central zone at E15.5 (Fig. 1f–h). Similar results were obtained with immunostaining using anti-Aldh1a3 antibodies (Supplementary Fig. 2). As the sensory epithelium matures, Aldh1a3 immunoreactivity is localized to both HCs and SCs in the extrastriola and some HCs in the striola by postnatal day 0 (P0) (Supplementary Fig. 2). Overall, these expression patterns suggest that differential expression of RA could establish the central and peripheral zones of vestibular organs.

### Formation of the striolar/central zone requires *Cyp26b1*

**Fig. 2:**
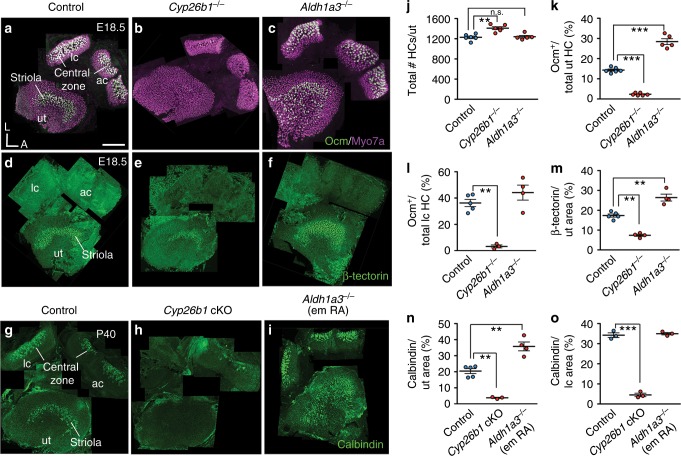
Disruption of RA signaling alters striolar/central zone formation. **a** Oncomodulin (Ocm) is expressed in type I HCs in striolar/central zones, whereas all HCs are positive for Myosin7a of controls. **b**, **c**, **j** Total HC number is increased in *Cyp26b1*^*−/−*^ (1394 ± 39, *P* = 0.014, *n* = 6), but unchanged in *Aldh1a3*^*−/−*^ utricles (1285 ± 28, *P* = 0.419, *n* = 5), compared to controls (1229 ± 30, *n* = 6). **b**, **c**, **k** Percentages of Ocm^+^ HCs (14.3 ± 0.5 in controls, *n* = 6) are decreased in *Cyp26b1*^*−/−*^ (2.3 ± 0.2, *n* = 6, *p* < 0.0001) and increased in *Aldh1a3*^*−/−*^ utricles (28.4 ± 1.4, *n* = 5, *P* < 0.0001). **b**, **c**, **l** Percentages of Ocm^+^ HCs in lateral cristae (lc, 36.2 ± 2.7 in controls, *n* = 5) are reduced in *Cyp26b1*^*−/−*^ (3.2 ± 1.1, *n* = 3, *P* = 0.0005), but unchanged in *Aldh1a3*^*−/−*^ mutants (44.1 ± 5.6, *n* = 4, *P* = 0.302). **d** Anti-β-tectorin labels SCs in striolas of utricles. **e**, **f**, **m** β-tectorin^+^ domain, as % of total utricular macular area (17.4 ± 0.9 in controls, *n* = 5), is reduced in *Cyp26b1*^*−/−*^ (7.4 ± 0.6, *n* = 4, *P* = 0.0002) and increased in *Aldh1a3*^*−/−*^ (26.4 ± 1.7, *n* = 4, *P* = 0.0003) utricles. **g** Anti-calbindin labels calyceal nerve endings in striolar/central zones. **h**, **i**, **n**, **o** Expression and percentage of calbindin-positive area is reduced in utricles (3.8 ± 0.3, *n* = 3, *P* = 0.0005) and cristae (4.7 ± 1.4, *n* = 3, *P* < 0.0001) of *Cyp26b1* cKO, but increased in utricles (36.0 ± 6.6, *n* = 4, *P* = 0.0006) and unchanged in cristae (34.4 ± 0.5, *n* = 3, *P* = 0.3021) of *Aldh1a3*^*−/−*^ (em RA) mutants, compared to respective controls (**n**, 19.7 ± 1.7, *n* = 5; **o**, 34.2 ± 1.1, *n* = 3). Error bars: SEM. Significance was assessed by one-way ANOVA for all panels. A, anterior; L, lateral. **P < 0.01 and ***P < 0.001. n.s., not significant. Scale bar: 200 μm.

To test the hypothesis that striolar/central zone formation in each vestibular sensory organ requires RA degradation, we analyzed vestibular organs in gain and loss of RA function mutants, *Cyp26b1*^*−/−*^ and *Aldh1a3*^*−/−*^ embryos, respectively, between E17.5 and E18.5, and these mutants survive until birth^29,30^. Total HC number is increased by 13% in *Cyp26b1*^*−/−*^ utricles, but unchanged in *Aldh1a3*^*−/−*^ utricles, compared to controls (Fig. 2j). The Ca^2+^-binding protein oncomodulin (Ocm) is expressed only in the type I HCs located within the striolar/central zone (Fig. 2a)^31^. In *Cyp26b1*^*−/−*^ inner ears, Ocm expression is reduced in both maculae and cristae (Fig. 2b, Supplementary Fig. 3). Percentages of Ocm^+^ HCs are decreased in *Cyp26b1*^*−/−*^ utricles (Fig. 2k). Similar decreases in Ocm + HCs were observed in saccules (Supplementary Fig. 3) and lateral cristae (Fig. 2l).

In *Aldh1a3*^*−/−*^ mutants, in which RA synthesis is reduced, the Ocm expression domain is increased in the maculae but not cristae (Fig. 2c, Supplementary Fig. 3). In *Aldh1a3*^*−/−*^ utricles, the expression domain of Ocm expands towards the medial extrastriolar (MES) region (Fig.1a), but not the lateral extrastriolar (LES) region of utricles (Fig. 2c, Supplementary Fig. 4); in *Aldh1a3*^*−/−*^ saccules, in contrast, the Ocm expression domain is broadly expanded (Supplementary Fig. 3). The percentages of Ocm^+^ HCs are increased per utricle (Fig. 2k) and saccule (Supplementary Fig. 3), compared to controls. In contrast, no increase in Ocm^+^ HCs is evident for the lateral crista (Fig. 2l), suggesting the presence of other compensatory sources of RA in the cristae. Nevertheless, these combined results (Table 1) suggest that formation of the striolar/central zone-specific HCs in vestibular organs is regulated by Cyp26b1-mediated degradation of RA.

**Table 1:** Phenotypic summary of *Cyp26b1* cKO and *Aldh1a3* (em RA) KO.

Striolar/Central Zone markers	Utricle		Saccule		Crista	
	*Cyp26b1* cKO	*Aldh1a3* KO	*Cyp26b1* cKO	*Aldh1a3* KO	*Cyp26b1* cKO	*Aldh1a3* KO
Ocm (HC)	**↓**	**↑**	**↓**	**↑**	**↓**	No change
β-tectorin (SC)	**↓**	**↑**	No change	No change	N/A	N/A
Calbindin (afferent)	**↓**	**↑**	**↓**	N/D	**↓**	No change

*N/A* not available

*N/D* not determined

We next examined whether the identity of SCs in the striolar/central zone of vestibular organs is also affected in mutants deficient for enzymes in the RA pathway. In normal maculae, β-tectorin is specifically expressed in striolar SCs (Fig. 2d, m)^28^. Consistent with the changes in Ocm expression by HCs, the extent of β-tectorin expression is reduced in *Cyp26b1*^*−/−*^ utricles (Fig. 2e, m) and increased in *Aldh1a3*^*−/−*^ utricles (Fig. 2f, m). These results show that striolar SC identity in the utricle requires the decrease in striolar RA levels that is mediated by Cyp26b1. In the saccule, in contrast, no significant difference in the β-tectorin-positive region was detected in either *Cyp26b1*^*−/−*^ or *Aldh1a3*^*−/−*^ ears (Table 1, Supplementary Fig. 3), suggesting that changes in RA levels may not affect SC formation in the saccule. In cristae, which have different accessory structures than the maculae, β-tectorin is not expressed (Fig. 2d) and there is no known SC marker for the central zone, preventing the evaluation of RA effects on SC identity.

Many regional differences in the vestibular organ develop postnatally. For example, osteopontin, an extracellular matrix protein, is found in extrastriolar type I HCs at birth^32^. Another prominent feature of mammalian vestibular epithelia is the postnatal development of afferent nerve endings (calyces) encasing single or multiple type I HC bodies (Fig. 1a)^8,11,12^. In order to investigate the role of RA in postnatal differentiation, we bypassed the lethality of *Cyp26b1*^*−/−*^ and *Aldh1a3*^*−/−*^ mice^29,30^ by first generating cKO of *Cyp26b1* (*Foxg1*^*Cre*^*;Cyp26b1*^*lox/−*^) using *Foxg1*^*Cre*^, which is expressed in tissues such as the otic placode and forebrain during early embryogenesis^33^. We also generated viable *Aldh1a3*^*−/−*^ mice by supplementing maternal RA during embryonic day (E) 8.5 to E14.5 of development (*Aldh1a3*^*−/−*^ (em RA)). This RA supplementation has been demonstrated to rescue the RA requirements of *Aldh1a3*^*−/−*^ mice during early embryogenesis without reducing the inner ear defects, which develop later^29,34^. In *Cyp26b1* cKO mice, reduction of Ocm and β-tectorin expression patterns are similar to those of the *Cyp26b1* KOs (Supplementary Fig. 5). Additionally, we investigated the nature of the Ocm^−^ type I HCs in the striola of *Cyp26b1* cKO utricles using anti-osteopontin antibodies, which labels extrastriolar type I HCs postnatally^32^. *Cyp26b1* cKO utricles lack normal striolar identifying features and we delineated a comparable region to the control striola (see Methods) for comparison to controls. Our results showed that there is an upregulation of osteopontin immunoreactivity in *Cyp26b1* cKO striolas (Supplementary Fig. 6). Together, these results suggest that the type I HCs are present in the *Cyp26b1* mutant striola, but they have acquired some extrastriolar type I HC properties.

Additionally, we used calbindin-D28K immunoreactivity to selectively stain for striolar/central zone afferents^35^ in control and mis-regulated RA mutants at P40 or P45. Similar to what was observed for HCs and SCs, the calbindin expression domain is decreased in all *Cyp26b1* cKO vestibular sensory organs compared to controls (Fig. 2g, h, n, o, Supplementary Fig. 3). These results indicate that the development of calyceal-bearing afferents of striolar/central zones requires relatively lower levels of RA signaling regulated by *Cyp26b1*.

In *Aldh1a3*^*−/−*^ (em RA) mice, the calbindin-expressing domain is increased in the utricle but not in the lateral crista (Fig. 2i, n, o, Table 1), consistent with the expanded striolar region in the *Aldh1a3*^*−/−*^ mutants. Together, these results suggest that formation of the striolar/central zone in the vestibular organs requires Cyp26b1 enzyme to degrade the RA emanating from the surrounding sensory tissue, which is largely generated by Aldh1a3 in the maculae, although not in the cristae (Table 1).

### *Cyp26b1* deletion affects anatomical features of the striola

**Fig. 3:**
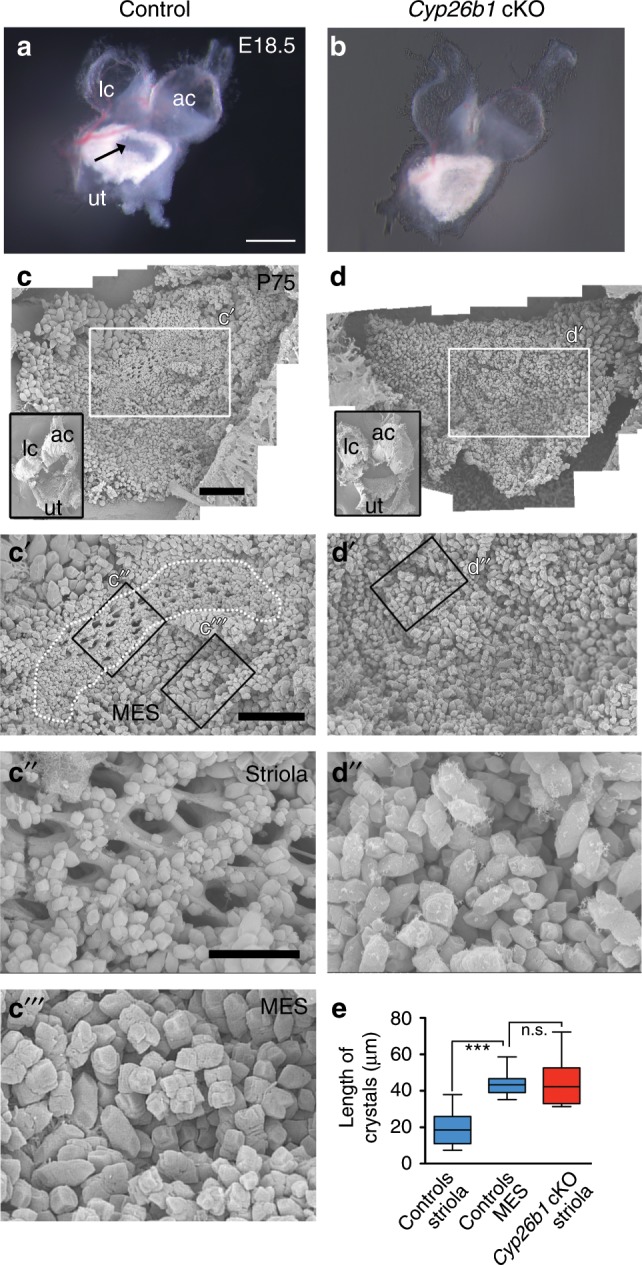
Loss of regional differences in the otoconia of *Cyp26b1* cKO utricles. **a**, **b** A dissected utricle (ut), anterior (ac), and lateral cristae (lc) of *Foxg1*^*Cre*^*;Cyp26b1*^*lox/+*^ control (**a**) and *Cyp26b1* cKO (**b**) ears at E18.5. In control utricle, the striola region shows a clearance of the otoconia (arrow in **a**), which is missing in *Cyp26b1* cKO utricles. **c**–**e** SEM of otoconia in *Foxg1*^*Cre*^*;Cyp26b1*^*lox/+*^ controls (**c**–**c‴**) and *Cyp26b1* cKO (**d**–**d″**) utricles at P75. Insets show low-power views of respective utricles. In controls (**c′**, **c″**, **c‴**), the striolar region (dotted outline) shows otoconia with smaller crystals (**c″**, **e**, 19.7 ± 1.5 μm, *n* = 22 crystals) and perforated holes in the subcupular meshwork layer (**c″**), whereas the medial extrastriolar region (MES) shows larger otoconia (**c‴**, **e**, 40.2 ± 2.2 μm, *n* = 23, *P* < 0.0001). There is no clear regional difference in the size of the otoconia in the *Cyp26b1* cKO mutant utricle (**d**, **d′**), and the otoconia crystals in the presumptive striolar region are larger in size (**d″**, **e**, 42.9 ± 3.1 μm, *n* = 21), comparable to those found in MES of controls (**c‴**, *P* = 0.6509). The one-way ANOVA with multiple comparisons was applied. In the box plots, bounds of box span from 25 to 75% percentile, center line represents the median, and whiskers represent the minimum and maximum of the data points. ***P < 0.001. Scale bars: 200 μm for **a**, **b**, 300 μm for **c**, **d**, and **c′**, **d′**, and 100 μm for **c″**, **c‴**, **d″**. n.s., not significant.

Next, we investigated the mature anatomy of the striola in *Cyp26b1* cKO utricles as a result of altered RA signaling during embryogenesis. A significant characteristic of the maculae is the presense of the otoconia, mineralized calcium carbonate crystals embedded above the otolithic membrane (Fig. 1a)^9^. Under low magnification, the otoconial layer in the utricle is more transparent in the striola than the rest of the organ due to the smaller size and number of otoconia (Figs. 1a and 3a). This clear zone is absent in *Cyp26b1* cKO utricles (Fig. 3b). Scanning electron micrographs (SEMs) of control utricles show smaller otoconia and more extensive perforations of the underlying meshwork otoconia in the striola (Fig. 3c–c″)^9^ compared to the MES (Fig. 3c‴). In *Cyp26b1* cKO utricles, SEM revealed no regional differences in otoconial crystals (Fig. 3d–d″), consistent with low-power brightfield images (Fig. 3b). Crystals throughout *Cyp26b1* cKO utricles were of similar size to control MES crystals (Fig. 3e), indicating that the zonal difference in otoconial size was absent in the *Cyp26b1* cKO utricles.

At the sensory epithelium level, the striola has a lower density of HCs (number per surface area) than in the rest of the sensory organ^4^, reflecting larger apical cell surfaces. To investigate whether this regional marker is also affected by RA signaling, the density of HCs in the striolar region was compared between controls and *Cyp26b1* cKO mutants. In *Cyp26b1* cKO utricles, which lack normal striolar identifying features, we delineated a comparable region to the control striola (see Methods) for comparison. In control utricles, HC density was lower in striola than in LES (Supplementary Fig. 7). This regional difference in HC density was not detected in mutant utricles (Supplementary Fig. 7). Striolar HC density in *Cyp26b1* cKO mutant epithelia was significantly higher than in control striola and comparable to HC density in control-LES.

In control utricles, the kinocilium length is shorter in the striola than extrastriola^10^. Consequently, the ratio of the length of the kinocilium to that of the tallest stereocilium (K/S ratio) is also significantly smaller in the striola than extrastriola^10^ (Supplementary Fig. 8). We therefore measured whether RA signaling affected these properties. For this analysis, we referenced hair bundle location to the line of polarity reversal (LPR) of hair bundles, which is closely associated with the striola in macular organs (Fig. 1a), and hair bundle orientation was identified by phalloidin staining of actin-filled stereocilia or anti-spectrin staining of the cuticular plate in which stereocila insert^36^. In the mouse, the utricular striola is largely medial to the LPR, whereas the saccular striola straddles the LPR (Fig. 1a, Supplementary Fig. 9)^37^. We determined that the relative position of the LPR was not altered in either the mutant utricle or saccule (Supplementary Fig. 9). In utricles, the K/S ratio in the *Cyp26b1* cKO striola was comparable to its value in the control extrastriola, but not the control striola (Supplementary Fig. 8). These results suggest that the zonal difference in the K/S ratio is absent in the *Cyp26b1* cKO utricles.

**Fig. 4:**
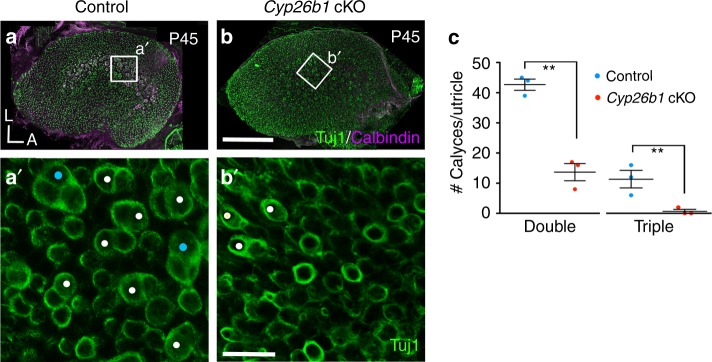
Pure/complex calyces are reduced in *Cyp26b1* cKO mice. **a**–**c** P45 whole-mount utricles from controls (**a**, **a′**) and *Cyp26b1* cKO (**b**, **b′**) immunolabeled with anti-Tuj1 (green) and anti-calbindin (magenta) antibodies. **a**, **b** Maximum intensity projection of the entire utricle. **a′**, **c** Enlarged, single plane image of the rectangular region in control striola (**a**), showing the presence of large number of double (white circles, 42.6 ± 1.8 double calyces/utricle) and triple (cyan circles, 10.0 ± 2.0 triple/utricle, *n* = 3 utricles) calyces at the cell body level. **b′**, **c** Fewer double calyces (white circles, 13.6 ± 2.8 double calyces/utricle, *P* = 0.001, unpaired *t* test) and no triple calyx (0.6 ± 0.6 triple calyces/utricle, *P* = 0.006, *n* = 3) are found in the corresponding region of *Cyp26b1* cKO mutants. Scale bars: 200 μm for **a**, **b**; 30 μm for **a′**, **b′**. **P < 0.01. A, anterior; L, lateral. Error bars: SEM.

Visual inspection of living utricular epithelia with differential contrast microscopy suggested that complex calyces, a prominent feature of the striolar/central zone^4,5,10^, were less numerous in *Cyp26b1* cKO utricles. To quantitatively examine this impression, we stained neurons with Tuj1 antibody and counted calyces in control and *Cyp26b1* cKO utricular striolas. Complex calyces encasing two or three type I HCs (double or triple calyces, respectively) were present in significantly higher numbers in control striolas (Fig. 4a, c) than in *Cyp26b1* cKO striolar regions (Fig. 4b, c).

Together, our results showed that the lack of *Cyp26b1* during embryogenesis affects many mature features of the striola, including the otoconia, expression of Ca^2+^-binding proteins, HC density, hair bundle morphology, and incidence of complex calyces, suggesting that striolar maturation depends on low levels of RA signaling.

### Striolar physiology is affected in *Cyp26b1* cKO mice

To assess whether zonal differences in physiology were affected by the mutation, we recorded from HCs and afferent calyces in both control and *Cyp26b1* cKO mice using a semi-intact preparation of the utricle and distal nerve. We visualized the epithelium’s apical surface with differential-interference-contrast optics, which allow resolution of hair bundles, HC bodies, and calyceal afferent terminals. Because the *Cyp26b1* cKO utricles had lost the zonal markers that we usually use (hair bundle size, HC density as viewed from above, the proportion of calyces that are complex), we relied on the LPR, assigning recordings in the first 10 HC rows medial to the LPR as “striolar.” We performed the whole-cell recording configuration on visually identified type I or type II HCs or on calyceal afferent terminals that surround type I HCs, and measured voltage-gated currents in voltage clamp mode and resting potential and step-evoked voltage changes in current clamp mode. Recorded HCs and calyceal terminals were filled with fluorescent dye, allowing their visualization with fluorescence optics.

In HCs (*n* = 64, from 21 mice), there were no obvious effects of the *Cyp26b1* cKO manipulation on whole-cell voltage-sensitive conductances, as revealed by currents evoked by voltage steps or voltage responses to current steps. Wild-type type I and type II cells from mice and other amniotes^38^ have outwardly rectifying K^+^ currents with very different voltage dependence: the voltage of half-maximal activation (*V*_1/2_) is 40–70 mV more negative in type I HCs compared to type II HCs. The unusually negative voltage dependence of the type I-specific K^+^ conductance (*g*_K,L_) has significant consequences for the size and speed of the receptor potential and appears to be essential for non-quantal transmission at type I–calyx synapses^39^. This key difference was preserved in the striolar zones of *Cyp26b1* cKO utricles: three-factor analysis of variances (ANOVAs) on *V*_1/2_ for HC type (I vs. II), genotype, and epithelial zone yielded a highly significant difference for HC type (*F*(1,46) = 1348.33, *P* = 0), but for no other comparison. Thus, the *Cyp26b1* cKO mutation did not grossly affect the electrophysiological differentiation of type I and type II HCs.

**Fig. 5:**
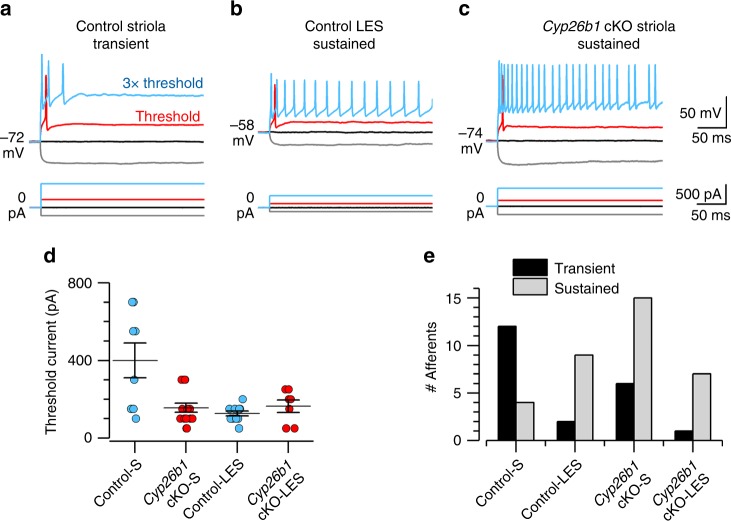
Neural activity in the striola of *Cyp26b1* cKO mice is extrastriolar-like. **a**–**d** *Cyp26b*1 cKO striolar afferents, like control extrastriolar afferents, were more excitable than control striolar afferents, as shown by their tendency to fire more spikes and by reduced current thresholds for spiking. **a**–**c** Whole-cell current clamp records from patched calyces of a control striolar (Control-S) afferent (**a**), control lateral extrastriolar (Control-LES) afferent (**b**), and a *Cyp26b1* cKO striolar (*Cyp26b1* cKO-S) afferent (**c**). Five hundred-millisecond current steps were delivered in 50-pA increments from –200 pA to >1 nA relative to zero holding current; a subset is shown including the response at threshold current (red). Three times (3×) threshold current- (blue) evoked transient spiking in the control striolar afferent, but sustained spiking in the control-LES afferent and *Cyp26b1* cKO-S afferent. **d** Mean threshold current to activate spikes was significantly higher in Control-S calyces than in all other categories—that is, the *Cyp26b1* cKO-S calyces resembled *Cyp26b1* cKO lateral extrastriolar (*Cyp26b1* cKO-LES) calyces and Control-LES calyces. Two-way ANOVA showed main effects of genotype (control vs. *Cyp26b1* cKO, *F*(1,41) = 6.1, *P* = 0.02), zone (striola vs. LES, *F*(1,41) = 10.0, *P* = 0.003) and their interaction (*F*(1,41) = 11.2, *P* = 0.002). Control-S afferents (400 ± 90 pA, *n* = 8) differed significantly from each other category, including control-LES afferents (127.3 ± 12 pA, *n* = 11; *P* = 0.0003, effect size 1.9) and *Cyp26b1* cKO-S afferents (156.3 ± 23 pA, *n* = 16; *P* = 0.0005, effect size 1.6). *Cyp26b1* cKO-S afferents did not differ from control-LES afferents (*P* = 0.94) or *Cyp26b1* cKO-LES afferents (164 ± 32 pA, *n* = 7, *P* > 0.99). **e** Transient firing was more common than sustained firing in Control-S afferents; sustained firing was more common in Control-LES, *Cyp26b1* cKO-S, and *Cyp26b1* cKO-LES afferents. Error bars: SEM.

Differences were seen, however, when we compared spiking activity of striolar and LES calyx-bearing afferents in *Cyp26b1* cKO and *Foxg1*^*Cre*^*;Cyp26b1*^*lox/+*^ controls. In the LES, calyx-bearing afferents are dimorphic afferents (forming both calyx and bouton terminals, Fig. 1a). In the striola, calyx-bearing afferents are either dimorphic or pure-calyx afferents. We measured spiking activity of dimorphic afferents or pure-calyx vestibular afferents via ruptured-patch recordings from their calyceal terminals (spikes initiate on the afferent neurite below the base of the calyx^8^). Striolar afferent neurons were more excitable in *Cyp26b1* cKO mice compared to *Foxg1*^*Cre*^*;Cyp26b1*^*lox/+*^ controls, as measured by lower current threshold for spiking and tendency to fire a longer train of spikes in response to supra-threshold current steps. Threshold current was defined as the lowest current at which spiking occurred when currents were incremented in 50-pA steps (red traces, Fig. 5a–c). Threshold currents were significantly larger for striolar afferents than for control-LES afferents and any other afferent class, including *Cyp26b1* cKO striolar afferents (Fig. 5d). Threshold currents did not differ significantly between *Cyp26b1* cKO striolar afferents and *Cyp26b1* cKO-LES afferents or control-LES afferents. Thus, *Cyp26b1* cKO manipulation reduced threshold current for spiking in striolar afferents to levels typical for extrastriolar afferents.

Afferent firing patterns were classified for current steps 2–3 times threshold current as either transient (1–3 onset spikes) or sustained (>4 spikes). The tendency of isolated neurons to respond to injected current steps with transient or sustained responses correlates with their tendency to fire more irregularly or more regularly in vivo, when they are driven by synaptic inputs from HCs^15^. In control utricles, striolar afferents were more likely to have transient than sustained responses and extrastriolar afferents were more likely to have sustained than transient responses (Fig. 5e), consistent with previous data from rat vestibular ganglion neurons^15^ and calyceal terminals^40^. In *Cyp26b1* cKO utricles, the normal zonal difference in firing pattern disappeared: most striolar afferents, like extrastriolar afferents, had sustained responses to current steps (Fig. 5e).

These changes in excitability in the* Cyp26b1* cKO zone corresponding to the striola (reduced current threshold, more sustained firing) likely involve loss of striolar-specific ion channel expression. Candidates include low-voltage-activated K (K_LV_) channels, which reduce neuronal excitability by increasing K^+^ conductance around resting potential, such that more current is required to depolarize to spike threshold. In normal utricular afferents, K_LV_ channels are expressed more in striola than extrastriola^8,15,40,41^. *Cyp26b1* cKO afferents may lack this zonal difference in K_LV_ expression, based on measurements of K_LV_ current that is on at –65 mV and turned off by stepping membrane voltage to –125 mV. In Cyp26b1 cKO utricles, K_LV_ current per afferent calyx recording did not differ significantly across zones (striola: 870 ± 114 pA, *n* = 16; extrastriola:1192 ± 151 pA, *n* = 6; *P* = 0.14). Thus, *Cyp26b1* cKO utricles showed changes in afferent physiology that are consistent with a loss of striolar identity.

### Absent VsEP in *Cyp26b1* cKO mice

**Fig. 6:**
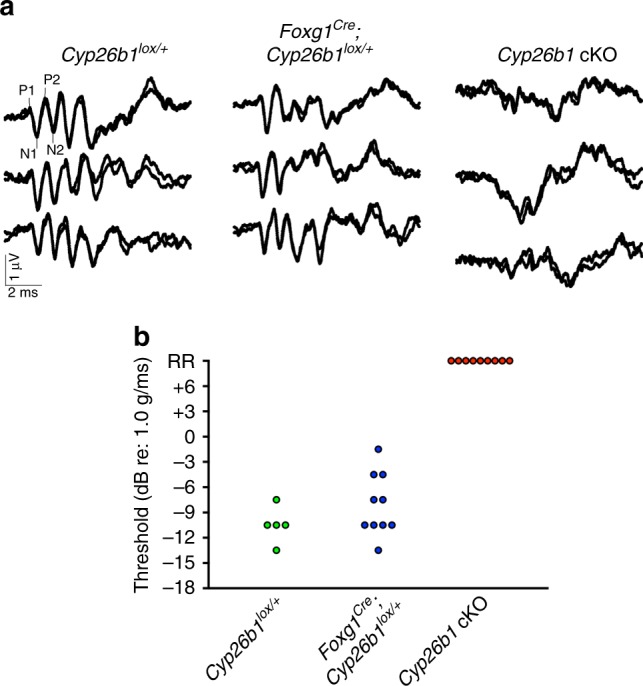
Absence of linear vestibular-evoked potential (VsEP) in *Cyp26b1* cKO mice. **a** Three representative VsEP waveforms for each genotype recorded at maximal jerk stimulus (+6 dB). In the *Cyp26b1* cKO mutants, distinct peaks of P1–P2 and N1–N2 are not detectable. **b** Summary of thresholds for VsEP determined by various jerk magnitudes. There was no significant difference in VsEP thresholds between the *Cyp26b1*^*lox/+*^ (−10.5 ± 1.34 dB re: 1 g/ms, *n* = 5) and *Foxg1*^*Cre*^*;Cyp26b1*^*lox/+*^ heterozygotes (−8.1 ± 1.17 dB re: 1 g/ms; *n* = 10, *P* = 0.1026, unpaired *t* test). *Cyp26b1* cKO mutants generated only a remnant response (RR).

To assess the functional consequences of losing the striola in vivo, we recorded with scalp electrodes the VsEP, which represent summed far-field afferent responses to transient linear accelerations, in control and *Cyp26b1* cKO mice. Control mice (*Cyp26b1*^*lox/+*^ and *Foxg1*^*Cre*^*;Cyp26b1*^*lox/+*^ heterozygous animals) had normal VsEP responses, whereas *Cyp26b1* cKO mice showed either absent or deficient VsEP responses (Fig. 6a). VsEP thresholds for *Cyp26b1*^*lox/+*^ and *Foxg1*^*Cre*^*;Cyp26b1*^*lox/+*^ heterozygotes were similar (Fig. 6b). There were also no differences in VsEP response activation latencies (P1, N1) or amplitude (P1–N1) at the highest stimulus level (+6 dB re: 1 g/ms) across the two genotypes. Taken together, these data show that VsEP was severely affected in *Cyp26b1* cKO mice, indicating that loss of the striola effectively abolishes most, if not all, VsEP response, supporting the hypothesis^2^ that the VsEP is a summated potential reflecting activity of striolar afferents and their downstream targets.

### Normal VOR and OVAR in *Cyp26b1* cKO mice

**Fig. 7:**
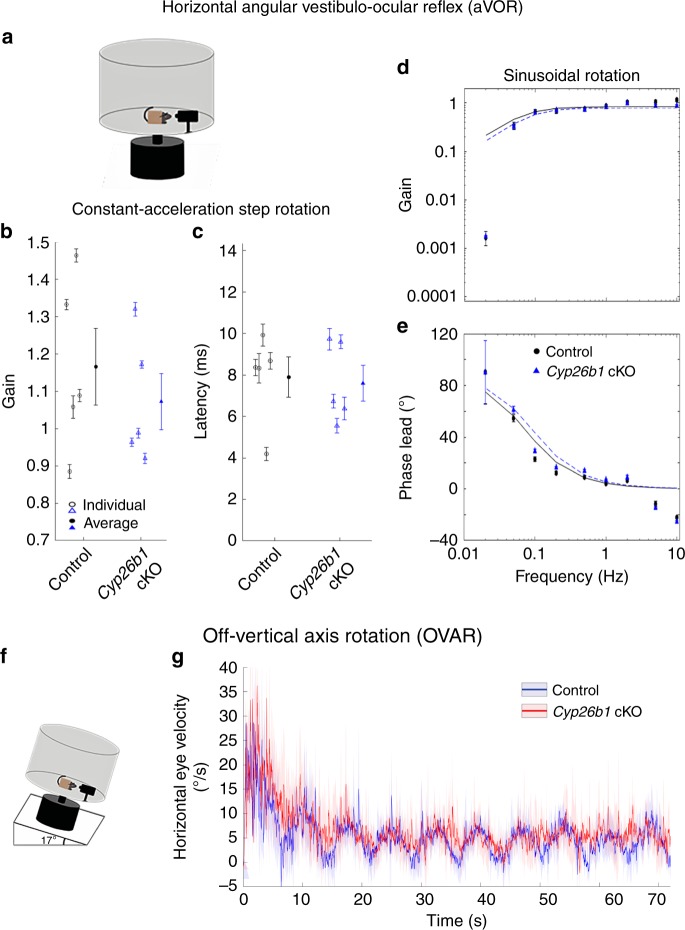
Normal aVOR and OVAR responses in *Cyp26b1* cKO mice. **a** Schematic view of aVOR apparatus. Constant-acceleration step gain *G*_A_ (**b**) and latency (**c**) for aVOR responses to whole-body, 3000°/s^2^ whole-body passive yaw rotations in darkness about an Earth-vertical axis through the head. Open markers denote individual mice; thick markers and lines show mean ± SEM. Gain (**d**) and phase lead (**e**) for yaw slow-phase aVOR responses to 0.02–10 Hz, 100°/s peak velocity sinusoidal, whole-body passive yaw rotations. Solid and dashed lines show first-order high-pass filter model fits to control and *Cyp26b1* cKO mouse population data, respectively. The high variability of phase and 0.02 Hz and relatively poor fit to gain data at 0.02 Hz are due to the small amplitude of responses at that frequency. Differences between control and *Cyp26b1* cKO mice were not significant for *G*_A_, latency, model gain, or model time constant (*P* < 0.05 for all comparisons, Mann–Whitney *U* test). **f** Schematic view of apparatus for off-vertical axis rotation (OVAR). Pitch of the table was maintained at 17°. **g** No significant difference in the eye velocity was observed between control and *Cyp26b1* cKO mice during transient response (~20 s; amplitude: 43.8 ± 8.3 vs. 38.7 ± 7.4° (*P* = 0.66, unpaired *t* test), time constant: 4.1 ± 1.1 vs. 5.1 ± 2.3 s (*P* = 0.68, unpaired *t* test), when both semicircular canal and otolith organs are stimulated, and steady-state response (~20 s; amplitude: 5.3 ± 0.72 vs. 5.8 ± 0.75° (*P* = 0.64, unpaired *t* test), bias: 10.5 ± 1.3 vs. 8.5 ± 1.3 (*P* = 0.33, unpaired *t* test), time when only responses from otolith organs are expected to be measured.

Next, we tested whether the VOR was also affected in the *Cyp26b1* cKO mutants based on the hypothesis that striola and central zones are important for vestibular-reflex function^14,19^. The VOR plays an important role in ensuring gaze stabilization during everyday activities by producing compensatory eye movements in the direction opposite of the head movement^42^. Because the dynamic response properties (i.e., gain and phase) of the VOR can be precisely quantified, it has also become a valuable tool for assessing vestibular function in mice. The best characterized VOR is the aVOR, which counter-rotates eyes in the horizontal plane and is largely driven by signals from horizontal canal cristae. As a control for eye muscle function, we also examined the optokinetic reflex (OKR), which uses visual signals to control eye motion, allowing tracking of a moving visual scene or objects. Normally, OKR functions in concert with VOR and vestibular neck reflexes to achieve the correct head and eye motion to stabilize visual field^42^. Both the *Foxg1*^*Cre*^*;Cyp26b1*^*lox/+*^ heterozygotes and *Cyp26b1* cKO mice showed robust OKR responses. Although aVOR gain increased and aVOR phase decreased systematically with increasing frequency of stimulus, there were no significant differences between *Foxg1*^*Cre*^*;Cyp26b1*^*lox/+*^ heterozygotes and *Cyp26b1* cKO mice in gain, phase change, model gain, or model time constant up to 10 Hz (Fig. 7a–e; *P* > 0.05 for all comparisons).

Next, to detect reflex responses derived from macular organs, OVAR testing was conducted^43^. For this purpose, we analyzed the recorded eye movements from the mice with heads fixed to a rotating platform (50°/s constant velocity), tilted 17° with respect to the ground (Fig. 7f). The eye velocity comprises two different responses: (1) a transient canal-mediated response that initially increases then gradually decays and (2) an otolith-mediated steady-state response in which eye velocity oscillates around a constant bias with a sinusoidal waveform. We quantified differences between the two groups by comparing the amplitudes and time constants of transient responses and the amplitudes and biases of steady-state responses. Similar to aVOR results, both transient and steady-state OVAR responses were comparable for *Cyp26b1* cKO vs. controls (Fig. 7f, g). Together, these results indicate that despite the loss of striolar/central zone identity in vestibular sensory organs, horizontal aVOR and OVAR responses were normal by these assays in the *Cyp26b1* cKO mice, at least up to the maximal testable frequencies, which largely span the physiologically relevant range of head motion for mice in their natural environments^44^.

### Vestibular balance tests of *Cyp26b1* cKO mice

**Fig. 8:**
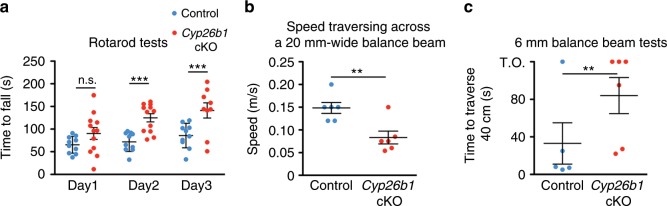
Impaired coordination of *Cyp26b1* cKO mice on balance beam. **a** Quantification of rotarod tests. Each mouse was placed on a rotating rod, which accelerated from 5 to 40 r.p.m. over a 5-min period. *Cyp26b1* cKO and *Foxg1*^*Cre*^*;Cyp26b1*^*lox/+*^ controls exhibited similar motor performance on day 1 of the trial (90.3 ± 15.9 s in mutants, *n* = 10, vs. 65.3 ± 6.5 s in controls, *n* = 10, *P* = 0.2573). *Cyp26b1* cKO mutants were able to stay on rod longer than controls on the second day of testing (123.3 ± 11.0 s mutants vs. 71.7 ± 7.5 s controls, *P* = 0.0003) and third day (141.2 ± 17.8 s mutants vs. 85.7 ± 9.5 s controls, *P* = 0.0037). The two-way ANOVA with multiple comparisons was applied. **b** Quantification of speed to traverse 60 cm distance on a 20-mm-wide beam. *Cyp26b1* cKO mutants moved slower (0.083 ± 0.014 m/s, *n* = 6) than controls (0.148 ± 0.011 m/s, *n* = 5, *P* = 0.0056, unpaired *t* test). **c** Quantification of time taken to cross 40 cm distance on a 6-mm-wide beam. Half of *Cyp26b1* cKO mutants failed to traverse the beam in 2 min (3/6), whereas most of controls reached the endpoints within 30 s (4/5). By assigning 2 min for all the mice that failed to complete the 40 cm distance, *Cyp26b1* cKO mutants moved slower (taking 84.0 ± 19.24 s to traverse the beam) than controls (33.0 ± 22.04 s for controls, *P* = 0.0099, unpaired *t* test). Error bars: SEM. **P < 0.01 and ***P < 0.001. TO, time out; n.s., not significant.

The vestibular system plays a vital role in balance control. To ensure stable body posture, VSRs produce compensatory movements of the neck and body to maintain the head in an upright position. Thus, we next tested *Cyp26b1* cKO mutants on a number of vestibular tasks used to test for balance and postural defects in mice. First, we found that these mice did not exhibit either circling or head tilting behaviors that are often associated with loss of vestibular function in rodents. Additionally, open-field testing revealed no hyperactivity in *Cyp26b1* cKO mutants (Supplementary Video 1, Supplementary Fig. 10), and the swimming ability of mutants was not affected under either light or dark conditions. We further subjected animals to rotarod testing^45^ and quantified their performance based on the time the animal remained on the rotating rod with increasing acceleration. *Cyp26b1* cKO and *Foxg1*^*Cre*^*;Cyp26b1*^*lox/+*^ controls exhibited similar motor performance on day 1 of the trial (Fig. 8a). However, *Cyp26b1* cKO mutants performed better (took longer time to fall) than controls on the second and third days (Fig. 8a). Whether this improved performance on the rotarod is due to replacement of striolar/central zone with extrastriolar/peripheral zone tissue or other mechanisms is not clear. Nevertheless, these collective behavioral results support the conclusion that the *Cyp26b1* cKO mutants exhibit no balance deficits during many of the tests commonly used to access balance and motor control functions in mice.

The balance beam test can detect subtle deficits in motor skills and balance that may not be detected by other standard motor and balance tests such as the rotarod test^46^. Accordingly, we tested the performance of *Cyp26b1* cKO mice on the balance beam. We found that *Cyp26b1* cKO mice walked slower than controls on a 20-mm-wide beam over 60 cm length (Fig. 8b). On a narrower beam (6 mm) over a distance of 40 cm, *Cyp26b1* cKO mice tended to freeze and stop walking (3 out of 6 vs. 1 out of 5 in controls) or moved slower than controls (Fig. 8c; Supplementary Video 2). These results indicate that *Cyp26b1* cKO mice show a deficit in coordinating challenging vestibulomotor functions.

### Increased head tremor in *Cyp26b1* cKO mice

**Fig. 9:**
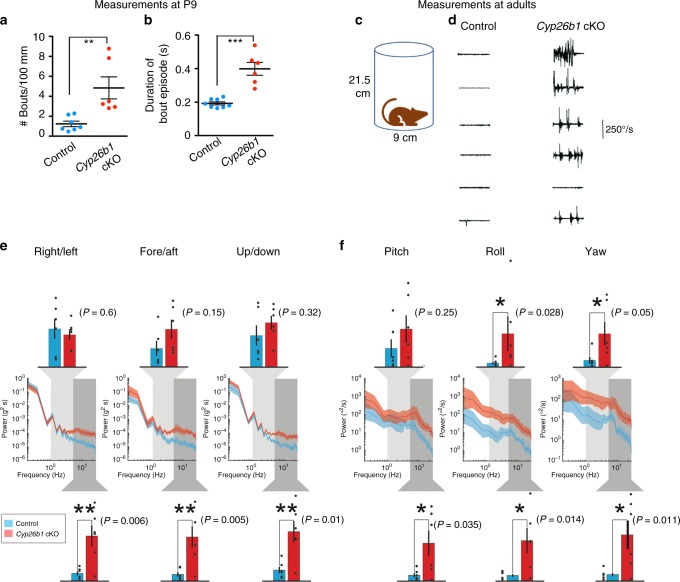
Increased head tremor in *Cyp26b1* cKO mice. **a**, **b** Quantification of head tremor for P9 pups. The number of bouts normalized over a 100 mm distance traveled (**a**) and average duration of each head-tremor episode over a 10-min period are shown (**b**). *Cyp26b1* cKO pups showed more frequent (**a**, 4.8 ± 1.1 in mutants, *n* = 6, vs. 1.2 ± 0.2 in controls, *n* = 8, *P* = 0.0059) and longer duration (**b**, 398 ± 38 ms in mutants vs. 192 ± 9 ms in controls, *n* = 8, *P* < 0.0001) head tremor, compared to *Foxg1*^*Cre*^*;Cyp26b1*^*lox/+*^ controls. **c** Schematic of the apparatus for measuring head tremor of an adult mouse at rest with a miniture head motion sensor affixed on the top of the skull. **d** *Cyp26b1* cKO showed characteristic head tremor (5/6), which were not present in controls. **e**, **f** Comparison of power spectra density of head movements in translational axes (**e**) and rotational axes (**f**) between controls (blue) and *Cyp26b1* cKO mutants (red). *Cyp26b1* cKO exhibit siginificantly higher power than controls at high frequencies (5–20 Hz, *P* = 0.006 for right/left, *P* = 0.005 for fore/aft, *P* = 0.01 for up/down, *P* = 0.035 for pitch, *P* = 0.014 for roll, and *P* = 0.011 for yaw axis, *n* = 6/group). Angular head velocity for yaw and roll axes of *Cyp26b1* cKO also had significantly higher power for frequencies between 1 and 5 Hz (upper graphs, *P* = 0.05 for yaw and *P* = 0.028 for roll axis). Unpaired *t* test was applied for all panels. Error bars: SEM. *P < 0.05, **P < 0.01, and ***P < 0.001.

Despite normal aVOR, OVAR, and performance for all standard motor and balance tests other than balance beam testing, we observed that it was possible to distinguish *Cyp26b1* cKO mice from controls in their cages, because the former demonstrated head tremor. This distinctive feature of *Cyp26b1* cKO mice was particularly obvious at early postnatal ages (Supplementary Video 3). Quantitative analysis at P9 showed that the incidence of head tremor per 100 mm distance traveled was higher in mutants than controls (Fig. 9a) and each head-tremor episode also lasted longer in mutants (Fig. 9b). These head tremor became less noticeable during normal activities in a cage as the mutants matured (Supplementary video 1), suggesting some level of compensation. To quantify head tremor in adult *Cyp26b1* cKO mutants, we affixed a 6D MEMS module consisting of three gyroscopes and three linear accelerometers to the mouse’s head implant. We confirmed that, when mice were not actively moving through their environment, *Cyp26b1* cKO adults exhibit head tremor (Fig. 9c, d). Power spectra of head movements revealed higher power for *Cyp26b1* cKO mutants than controls at high frequency (5–20 Hz) for all six dimensions (Fig. 9e, f). For mid-frequency (1–5 Hz), *Cyp26b1* cKO mutants have higher power for yaw and roll head velocity (Fig. 9f). Taken together, these results indicate that the ability to maintain head stabilization in *Cyp26b1* cKO mice is compromised.

## Discussion

Based on molecular, cellular, and physiological evidence, we demonstrated that striolar/central zones of vestibular sensory organs in the *Cyp26b1* mutants (gain of RA signaling) are severely reduced. We concluded that reduced RA signaling mediated by the expression of *Cyp26b1* in the prospective striolar/central zones is required for this regional formation. This conclusion is supported by results from a complementary mouse model, *Aldh1a3*^*−/−*^ mice, in which endogenous RA signaling is reduced in the peripheral region of sensory organs and striolar HCs are expanded in the two maculae (Fig. 2, Supplementary Fig. 3). The expansion of Ocm^+^ HC in the central zone is not observed in the cristae (Fig. 2c). We attributed this difference between maculae and cristae to possible functional redundancy provided by *Aldh1a1* and *Aldh1a2*, which are expressed in the adjacent nonsensory tissues or in the roof of the sensory organs^47^. Additionally, the extent of expansion of Ocm^+^ HCs is different between the utricle and saccule (Fig. 2c and Supplementary Fig. 3). Therefore, it is possible that intrinsic molecular differences across different regions of a sensory organ could also affect the response to the loss of RA signaling.

It is unclear how lower RA signaling mediates the unique cellular and anatomical features of the central regions during development. *Cyp26b1* is not detectable in the developing vestibular ganglion at E13.5 and P0 using in situ hybridization. Based on the concentrated expression of *Cyp26b1* in SCs of striolar/central zones, one possibility is that Cyp26b1 in SCs degrades RA and provides a niche for the afferent nerve endings to form calyces. Receptors of RA are known to be expressed in the otic vesicle and neural-sensory epithelium of the inner ear^48–50^. Alternatively, the reduced RA in striolar SCs could regulate downstream genes within SCs, which indirectly affect the development of HCs and their afferents. Previous results have suggested such a function for SCs^51^. Activated erbB receptors in SCs of the utricle lead to production of BDNF, which promotes synaptogenesis between HCs and afferent neurons.

VsEP measure utricular and saccular function and are produced in response to transient changes in linear head acceleration (jerk)^20,21^. Several lines of evidence have implicated the irregular afferents of the striola in the generation of VsEP. First, the phase-locking and transient firing properties of irregular neurons render them the most likely candidates to respond in a synchronized manner to linear jerk stimuli and produce the signature compound action potentials^2,19^. Second, VsEP is impaired by inhibitors of KCNQ (Kv7) channels, which are most abundant in the calyceal endings of the striola^18^. Third, the linear pulse stimuli used to evoke VsEP are likely to activate irregular, striolar, afferents based on experiments with sound and bone vibration stimuli in the 500–1000 Hz range^19,52^.

Our results provide strong evidence that the VsEP originates in the striola. The loss of morphological and physiological features in the striola of *Cyp26b1* cKO mice together with the loss of VsEP suggests that the striola is necessary for the generation of VsEP. In control otolith organs, mechanical input to HCs in different zones is differentially shaped by striking differences in otoconia, otolithic gel layers, hair bundle morphology, and bundle coupling to the otoconia^9^. These differences are likely to contribute to the greater sensitivity of striolar afferents to high-frequency and transient head motion, including the linear pulse stimuli used to evoke VsEPs. In *Cyp26b1* cKO mice, otoconia are present but have extrastriolar-like properties throughout the epithelium. Other likely factors are the zonal differences in calcium-binding proteins, afferent calyceal synapses, and excitability, all absent in *Cyp26b1* cKO.

Given the larger cell bodies, higher conduction velocities^53,54^, and greater phase advances of evoked firing^55–57^ of irregular neurons in striolar/central zones than regular neurons in extrastriolar/peripheral zones, these regions have been postulated to be important for mediating short-latency vestibular reflexes^58^. The aVOR in mice is fast with a latency as low as <7 ms^59^. However, we did not detect any deficit in aVOR and OVAR in *Cyp26b1* cKO mutants when lateral cristae and macular organs, respectively, were inertially stimulated, suggesting that the striolar/central zones of vestibular organs (innervated by irregular afferents) are dispensable for these functions, at least in the adults. We cannot rule out the possibility that vestibular reflexes at a younger age or stimulations beyond frequencies tested (10 Hz in the case of aVOR) may require normal function of the central zones. These results further suggest that regular afferents concentrated in the extrastriolar/peripheral zones of vestibular organs are important for mediating VOR. It was reported that *Aldh1a3*^*−/−*^ (em RA) mice show absence of aVOR and OVAR responses^34^, and our results show that the striolar/central zones in the vestibular organs are expanded based on Ocm staining (Fig. 2). Thus, these results are consistent with previous evidence that extrastriolar/peripheral zones are more important than striolar/central zones for aVOR and OVAR—for example, when irregular afferents in squirrel monkeys were selectively affected with galvanic currents, aVOR was not changed^60^. Together, these results suggest that regular afferents in extrastriolar/peripheral zones rather than irregular afferents in striolar/central zones are more important for mediating angular and linear VOR in mice.

A long-standing hypothesis is that irregular afferents, which innervate the striolar/central zone, preferentially contribute to VSR^61,62^. Our finding that loss of striolar/central zones leads to a deficit in coordinating challenging postural control on the balance beam is consistent with this proposal^61,62^. Surprisingly, however, *Cyp26b1* cKO mice performed well on other less sensitive tests commonly used for assessing changes in balance and motor control function in vestibularly deficient mice. Further, we did not observe common vestibular behavioral deficits such as head tilt and circling in the *Cyp26b1* cKO mutants. Together, these two observations suggest that the extrastriolar/peripheral zones, which constitute 75–80% of the sensory epithelia^4–6,13^ and are innervated by regular afferents, play a critical role in vestibulo-spinal as well as vestibulo-ocular reflexes in mice. In support of this hypothesis, regular afferents with their sustained firing properties are thought to be more important in conveying head positional information in steady state such as head tilts^63^.

A distinguishing characteristic of *Cyp26b1* cKO mice was that they demonstrated head tremor. Similar head-movement behaviors have been reported in both primates and humans with compromised vestibular function. For example, squirrel monkeys show transient head tremor and postural instability after plugging one of the lateral semicircular canals^64^. Patients with chronic bilateral vestibular loss who exhibit gaze variability and oscillopsia also show unusually pronounced head oscillations in response to weighted head mass^65^. These head tremors are attributed to failure of vestibular input that is normally required to maintain head stability^66,67^ and ensure movement accuracy during active goal-directed behaviors^65,68^. It is tempting to speculate that in the *Cyp26b1* cKO mouse, abnormal vestibular inputs from striolar/central regions cause head tremor, and affect challenging activities that require rapid vestibular input to motor centers, such as traversing a narrow balance beam. This phenotype is consistent with the postulated temporal precision of head-movement detection by the striolar/central zones^7,16^. One caveat is that *Cyp26b1* is also expressed in the cortex and brainstem^69^, and *Cyp26b1* cKO mice show some loss of expression in the brain, raising the possibility that the behavioral phenotype reflects central in addition to peripheral *Cyp26b1* loss.

In summary, using a genetic approach we generated a viable mouse mutant that largely lacks the striolar/central zone of vestibular organs. By disrupting RA signaling during embryogenesis, the entire axis of this specialized zone failed to develop properly, including associated components such as the otoconia and innervating neurons. The loss of this highly specialized and conserved region^70^ selectively affected the ability of vestibular afferents to respond to transient changes in linear acceleration, as shown by VsEP recordings, and produced some behavioral effects manifested as head tremor and balance beam deficits. A better understanding of the striolar/central zone-specific function has clinical and therapeutic relevance as HCs in this region are more susceptible to ototoxic insults in animal models^71^.

Low levels of RA signaling mediated by two of the RA degradation enzymes, Cyp26a1 and Cyp26c1, are also required for the formation of the retinal fovea^25^. Similar to *Cyp26b1* cKO effects on the entire striolar/central zone axis (accessory structure–HC–neurons), perturbation of RA signaling in the developing retina disrupts multiple foveal features, including photoreceptor distribution, ganglion cell density, and interneuron organization. Therefore, the same developmental strategy generates regional specialization and functional diversity in two sensory systems.

## Methods

### Mice and genotyping

The following mouse strains were used in the study: *Aldh1a3*^*+/−*^ (maintained in a mixed C57BL/6J and CD1 background)^72^, *Cyp26b*^*flox/flox*^ (RIKEN BRC (RBRC04333), maintained in a C57BL/6J background), and *Foxg1*^*Cre*^ ^33^ (RRID:IMSR_JAX:004337, maintained in C57BL/6J background). In addition, *Cyp26b1*^*+/−*^ mice were generated by breeding *Cyp26b1*^*flox/flox*^ mice with a ubiquitous cre line, *Actin*^*Cre*^, and maintained in a C57BL/6J background after recombination and removal of the *cre* allele. *Foxg1*^*Cre*^*;Cyp26b1*^*flox/−*^ were produced by crossing *Foxg1*^*Cre*^*;Cyp26b1*^*+/−*^ males with *Cyp26b1*^*flox/flox*^ females. Genotyping for mouse strains used in this study was conducted by Transnetyx Inc., based on the primers listed in Supplementary Table 1. All animal experiments were conducted under the approved animal protocols at the NIH, University of Chicago, University of Nebraska -Lincoln, and Johns Hopkins University, and according to NIH animal user guidelines.

### Tissue preparation

Timed pregnant females or postnatal mice were harvested and whole heads were hemi-sected, brain is removed, and fixed in 4% paraformaldehyde overnight. For anti-osteopontin staining, the specimens were fixed in Glyo-Fixx (Thermo Scientific) overnight. Then, half-heads were washed, cryo-preserved, and stored in −80 °C until processed for cryo-sectioning or whole-mount dissection subsequently.

### In situ hybridization

In situ hybridization was conducted as previously described^73^. Digoxigenin-labeled RNA probes were generated for *Cyp26b1* (GenBank: AW049789), β-tectorin^28^, and *Aldh1a3*^74^ as described.

### Whole-mount immunohistochemistry

Dissected saccules or utricles with anterior cristae and lateral cristae attached were blocked with PBS containing 4% normal donkey serum and 0.2% Triton X (PBT). Then, specimens were treated with primary antibodies diluted with blocking solution overnight at 4 °C. The primary antibodies used were as follows: goat polyclonal anti-Ocm (1:300, Santa Cruz Biotech, #sc-7446), rabbit polyclonal anti-β-tectorin (1:1000, a gift from Guy Richardson, Universtiy of Sussex), rabbit polyclonal anti-Myosin7a (1:1000, Proteus, #25-6790), mouse monoclonal anti-Myosin7a (1:50, Santa Cruz, sc-74516), rabbit polyclonal anti-calbindin (1:1000, Millipore, #AB1778), mouse monoclonal anti-βIII-tubulin (1:500; R&D, #MAB1195), mouse anti-βII spectrin (1:500; BD Bioscience, #612562), rabbit polyclonal anti-αII spectrin (1:500; Invitrogen, #PA5-35383), goat polyclonal anti-Jag1 (1:200; Santa Cruz, #sc-6011), goat polyclonal anti-Sox2 (1:500; Santa Cruz Biotech, #sc-17320), mouse monoclonal anti-Sox2 (1:150; Santa Cruz, sc-365823), goat polyclonal anti-osteopontin (1:500; R&D Systems, #AF808), rabbit polyclonal anti-Aldh1a3 (1:150; Millipore, #ABN427), and mouse monoclonal anti-acetylated tubulin (1:1000; Sigma-Aldrich, #T6793). Alexa Fluor 647-conjugated phalloidin was used to label actin-based stereocilia (Thermo Fisher Scientific, #A22287).

Following primary antibody incubation, samples were washed with PBT extensively before incubating with appropriate secondary antibodies conjugated with fluorescent proteins: donkey anti-mouse, rabbit, or goat IgG (H + L) antibody (Thermo Fisher Scientific) for 1 h at 4 °C. Then, samples were washed extensively with PBT before mounting with ProLong Gold Antifade (Invitrogen) and imaged with a Zeiss LSM780 confocal microscope. All low-power immunostaining pictures are composite of images taken at ×40 magnification.

### Scanning electron microscopy

The preparation of samples for SEM was conducted as described^75^. Briefly, the utricles and cristae were quickly dissected from harvested animals and submerged in fresh fixative consisting of 2.5% glutaraldehyde (Electron Microscopy Sciences), 2% formaldehyde (EMS), 3 mM calcium chloride, and 0.1 M cacodylate. Two hours after fixation at room temperature, the otoconia was exposed and then tissues were post fixed with osmium-thiocarbohydrazide-osmium method. Specimens were then dehydrated with a series of ethanol, followed by critical point drying. After spattering with platinum for coating, pictures were taken using electron microscope (SU4800, Hitachi).

### Measurement of otoconial size

SEM images were used to measure the length of each otoconia in control and mutant utricles (*n* = 2 for each genotype). To identify the central region in *Cyp26b1* cKO, the striola was identified in control utricles based on the smaller size of the otoconia. Then, a comparable region in the two mutant utricles was compared to that of the controls. The length of the otoconial crystals in the middle of striolar (4 mm^2^) and MES region were measured by using the ImageJ (1.52 h) software.

### Measurement of cilia length and K/S ratio

P3–P10 utricle samples (*n* = 4 for each genotype) were fixed and labeled with conjugated phalloidin, anti-acetylated tubulin (for kinocilium), and anti-αII spectrin antibodies. A composite picture of confocal images taken at ×40 magnification was generated and LPR was drawn based on hair bundle orientations. Then, regions lateral (lateral extrastriola) and immediately medial (striola) to the LPR in the center of the utricle were selected for stacked confocal images taken at the thickness of 0.2 μm. Length of the kinocilium and the tallest stereocilium were measured using the slice function of the Imaris software. Ratio of the height of the kinocilium (K) to the height of the tallest stereocilia (S) was calculated for 3–5 HCs in each region.

### RA treatment of timed pregnant females

Viable *Aldh1a3*^*−/−*^ mice was generated as described^29^. Briefly, RA powder (Sigma) was suspended in ethanol (5 mg/ml), and then 1 ml of RA solution was mixed with 50 mg of normal chow (5015, LabDiet) and administered to pregnant females ad libitum from E8.5 to E14.5.

### Measurement of HC density and osteopontin-labeled HCs

HC density in the utricle was measured as described^37^. Briefly, a straight line across the widest region of an utricle was drawn along the anterior–posterior (A–P) axis. Then, two lines perpendicular to the A–P line, which mark the middle-third region of the utricle were drawn. This middle region was divided into two equal halves along the medial–lateral axis, and the posterior half was further sub-divided into four equal regions marked as 1, 2, 3, and 4, representing LES, striola, and two MES regions, respectively (Supplementary Fig. 7a). The number of HCs per 0.01 mm^2^ area within areas 1 and 2 were counted.

### Quantification of complex calyces

Whole-mount utricles of *Cyp26b1* cKO and littermate controls at P30–45 were immunolabelled with anti-Tuj1 and anti-calbindin antibodies (*n* = 3/group). Z-stacked images were taken using a laser scanning confocal microscope (Zeiss LSM780). Tuj1-positive calyces that surround two or three HC bodies in each utricle were scored manually by examining individual confocal stacks.

### Whole-cell patch clamp recordings

Data shown here are from 21 littermates: 11 control mice (*Foxg1*^*Cre*^*;Cyp26b1*^*lox/+*^, ages P17–P97, median P21) and 10 mutant mice (*Cyp26b1* cKO, *n* = 10, P12–P100, median P20.5). Tissue preparation was conducted as described^40^. For each electrophysiological experiment, a mouse was anesthetized deeply by exposure to isoflurane and decapitated. The utricle plus the superior division of the vestibular ganglion and distal part of the vestibular nerve were excised, trimmed, and secured in a recording chamber with the exposed apical surface of the epithelium facing up, as described^40,76^.

Whole-cell recordings were made at room temperature (23–25 °C) using pipettes with resistances between 3 and 7 MΩ in standard solutions. The bath (external) solution was Leibovitz-15 (L15) medium, supplemented with 10 mM HEPES, ~315 mmol kg^–1^ and pH 7.4. The pipette (internal) solution comprised (in mM): 135 KCl, 0.1 CaCl_2_, 3.5 MgCl_2_, 3 Na_2_ATP, 5 creatine phosphate (Na^+^ salt), 0.1 Na-cAMP, 0.1 Li-GTP, 5 EGTA, and 5 HEPES. The solution was brought to pH 7.3 and ~300 mmol kg^–1^ by adding ~28 mM KOH. Sulforhodamine 101 (1 mg/100 ml; Invitrogen) was added to the internal solution to label the recorded HC or calyx.

Whole-cell, GΩ-seal recordings were made from visually identified HCs or calyceal afferent terminals within the semi-intact epithelium as described^40^. The EPC-10 (HEKA) patch clamp amplifier was controlled by Patchmaster software (HEKA Electronik GmbH). In voltage clamp mode, we recorded currents evoked by iterated voltage steps with the amplifier’s 4-pole low-pass corner frequency at 6 kHz and a sampling interval of 5–25 µs. Series resistances ranged from 4 to 32 MΩ (mean 10.4 ± 0.6 MΩ, *n* = 57) and were compensated during recordings by 80.1 ± 0.3%. Capacitive currents were nulled on-line with Patchmaster. We also recorded voltages evoked by iterated current steps in current clamp mode. Potentials are corrected for a liquid junction potential of 4 mV, calculated with the JPCalc software^77^ as implemented by Clampex 10 (Molecular Devices). Cells were held at –64 mV (in voltage clamp) and resting potential (in current clamp), unless otherwise noted. For steady-state analyses of voltage-dependent properties, command potentials were corrected for series resistance errors.

Data were analyzed with OriginPro 2017 (OriginLab, Northampton MA). Results are presented as means ± SE. Comparisons were made with two-factor ANOVA, with one factor being genotype (control vs. mutant) and the other epithelial zone (striolar vs. extrastriolar), followed by Tukey’s means comparisons. Effect size was estimated by Hedge’s *g* statistic.

### VsEP measurements

VsEP was measured in *Cyp26b1*^*lox/+*^ (*n* = 5), *Foxg1*^*Cre*^*;Cyp26b1*^*lox/+*^ (*n* = 10) and *Cyp26b1* cKO (*n* = 9) mice. VsEP recordings were the same as methods published previously^78–80^. The first positive (P1) and negative (N1) response peaks of the VsEP waveform were scored for each intensity level. Thresholds (measured in dB re: 1.0 g/ms) were also obtained from the scored VsEP waveforms.

### aVOR measurements

We measured eye movements of alert *Foxg1*^*Cre*^*;Cyp26b1*^*lox/+*^ (*n* = 5) and *Cyp26b1* cKO (*n* = 5) mice in response to transient (3000°/s^2^ constant acceleration to peak/plateau velocity of 300°/s lasting 0.8–1.0 s, then −3000°/s^2^ deceleration for 100 ms to rest at 90° from the starting position) yaw, whole-body head rotations in darkness using a fiducial-tracking, binocular 3D video-oculographic method and data analysis procedure identical to that described in detail previously^81^, except that the image acquisition rate was performed using new cameras with higher image resolution and framerate (180 frame/s). Sinusoidal stimuli were 0.02–10 Hz at peak velocity 100°/s for ≥10 cycles per trial. Eye rotation data were converted to rotation vectors in head coordinates and analyzed as described previously^81^. Yaw (horizontal) head angular velocity data were inverted prior to gain calculation. For transient stimuli, we restricted analysis to slow-phase nystagmus responses during the 100 ms constant-acceleration time segment after onset of each stimulus. For each trial, response latency was computed as the time difference between the zero-velocity-intercept times for lines fit in a least-mean-square sense to eye and head velocity during the constant-acceleration stimulus portion of the stimulus. Using the slopes of the same fitted lines, we computed “constant-acceleration segment gain” *G*_A_ as the mean over all cycles of the ratio of eye acceleration to head acceleration. For sinusoidal stimuli, we removed quick phases and saccades manually prior to further analysis of slow-phase nystagmus data, for which ≥10 cycles per trial were averaged and used to compute gain and phase of the yaw eye angular velocity response relative to the head angular velocity stimulus. Positive phase lead denotes a rightward slow-phase nystagmus response leading a leftward head rotation stimulus. Sinusoidal frequency response data were further parameterized by fitting a first-order high-pass filter to each animal’s data. The resulting gain and corner frequency parameters were used for statistical comparison between mouse groups. Gain and latency data were analyzed using a Mann–Whitney *U* test with significance set at *P* *=* 0.05.

### OVAR measurements

*Foxg1*^*Cre*^*;Cyp26b1*^*lox/+*^ controls (*n* = 6) and *Cyp26b1* cKO mice (*n* = 6), ranging between 6 and 7 months old, were used. Techniques employed for measurement of eye movements during OVAR in alert mice were described elsewhere^43^. Briefly, recordings made after fixating mice on a rotating platform, which was tilted 17° with respect to the ground. Platforms speed was increased from 0 to 50°/s in 500 ms and maintained its constant velocity for 72 s (10 complete rounds) before being stopped. Eye movements were measured using video occular-graphy (iScan). Quick phases were identified as previously described^82^ and excluded from subsequent analysis. We then estimated the time constant of the OVAR slow-phase eye velocity response decay, as well as the amplitude and phase of its sinusoidal modulation using a linear regression appoach^83^.

### Open-field test

Three-month-old *Foxg1*^*Cre*^*;Cyp26b1*^*lox/+*^ control (*n* = 6) and *Cyp26b1* cKO mice (*n* = 5) were used. Each mouse was placed in an open arena. Two minutes after habituation, subject was video recorded for 5 min. Trace of each mouse was visualized followed by analysis with Topscan software (3.0, Clever Sys Inc.).

### Rotarod test

*Foxg1*^*Cre*^*;Cyp26b1*^*lox/+*^ controls (*n* = 10) and *Cyp26b1* cKO mice (*n* = 9), ranging from 2 to 5 months old, were used. Each mouse was placed on a motorized rotating rod (ROTA ROD, Panlab, Harvard Apparatus) that gradually accelerated from 5 to 40 r.p.m. in 5 min, and the time required for the mouse to fall off the rotarod was scored. Each mouse underwent tests for three consecutive days with 5 trials per day. First day was considered to be the training day. Averaged score for each day was processed for statistical analysis.

### Balance beam test

*Foxg1*^*Cre*^*;Cyp26b1*^*lox/+*^ control (*n* = 5) and *Cyp26b1* cKO mice (*n* = 6), ranging between 6 and 7 months old, were used. The balance beam apparatus consisted of a 60-cm-long and 20-mm-wide beam that was positioned 70 cm above ground with an escape box on one end. Walking speed was measured by recording the time the animal took to reach the escape box from the opposite end of the beam. When 6-mm-wide beam was used, mice were placed at the midpoint of the 80-cm-long beam and the time taken to reach the endpoint on either side (time to traverse 40 cm) was measured. Mice were scored ‘’time out” when failed to reach the endpoint in 2 min.

### Head-tremor measurements of P9 pups

P9 controls (*n* = 8) and *Cyp26b1* cKO (*n* = 6) pups were placed in an open arena. Their motor activities and behavior were video recorded for 10 min. The total distance traversed, the number of bouts per 100 mm traveled, and duration of each bout episode were measured using the Topscan software.

### Head-tremor measurements of adult mice

Control (*n* = 6) and *Cyp26b1* cKO mice (*n* = 6) between 6 and 7 months old were used. Mice were placed in a cylinder (9 cm diameter and 21.5 cm height) that limited their motion, so mice maintained their steady posture. Head movements in six dimensions were recorded using a miniature head motion sensor (MPU-9250, SparkFun Electronics, Niwot, CO) affixed on the top of the skull, which comprises a three-dimensional (3D) accelerometer (measures linear acceleration; right/left, fore/aft, and up/down) and 3D gyroscope (measures angular velocity: pitch, roll, and yaw). Data were acquired at 200 Hz using windows-based CoolTerm software. We then computed the power spectral densities (pwelch function, MATLAB, MathWorks) using Welch’s averaged periodogram with nfft = 128 and a Bartlett window (128 ms duration) for all six dimensions of movement.

### Statistical analysis

*T* test was used for comparison of two samples, and either one- or two-way ANOVA, followed by Tukey’s multiple comparison tests was used for more than two samples. Data distribution was assumed to be normal, but not formally tested. All statistics were conducted by Prism 7 (GraphPad Inc.), except in cases described separately. All data are shown as average ± SEM.

### Reporting summary

Further information on research design is available in the Nature Research Reporting Summary linked to this article.

## Supplementary information

Supplementary Information

Peer Review

Reporting Summary

Description of Additional Supplementary Files

Supplementary Video 1

Supplementary Video 2

Supplementary Video 3

## Data Availability

The data that support the findings of this study are available from the corresponding author upon reasonable request.

## Code availability

VsEP data were collected using custom software, which is available upon request. A collection of scripts used to analyze and parametrize mouse horizontal VOR for sinusoidal and impulse motions can be found in https://github.com/ayiotis/mouse-VOR.
