# Supplementary material for: Retinoic acid degradation shapes zonal development of vestibular organs and sensitivity to transient linear accelerations
Source: Nat Commun. 2020 Jan 2;11:63. doi: 10.1038/s41467-019-13710-4 (PMC6940366; doi:10.1038/s41467-019-13710-4)
Supplement: Supplementary file 1 — Supplementary Information [file 41467_2019_13710_MOESM1_ESM.pdf]

Supplementary Information

Retinoic acid degradation shapes zonal development of vestibular organs and sensitivity to transient linear accelerations

Ono *et al*.

Supplementary Figures and Legends

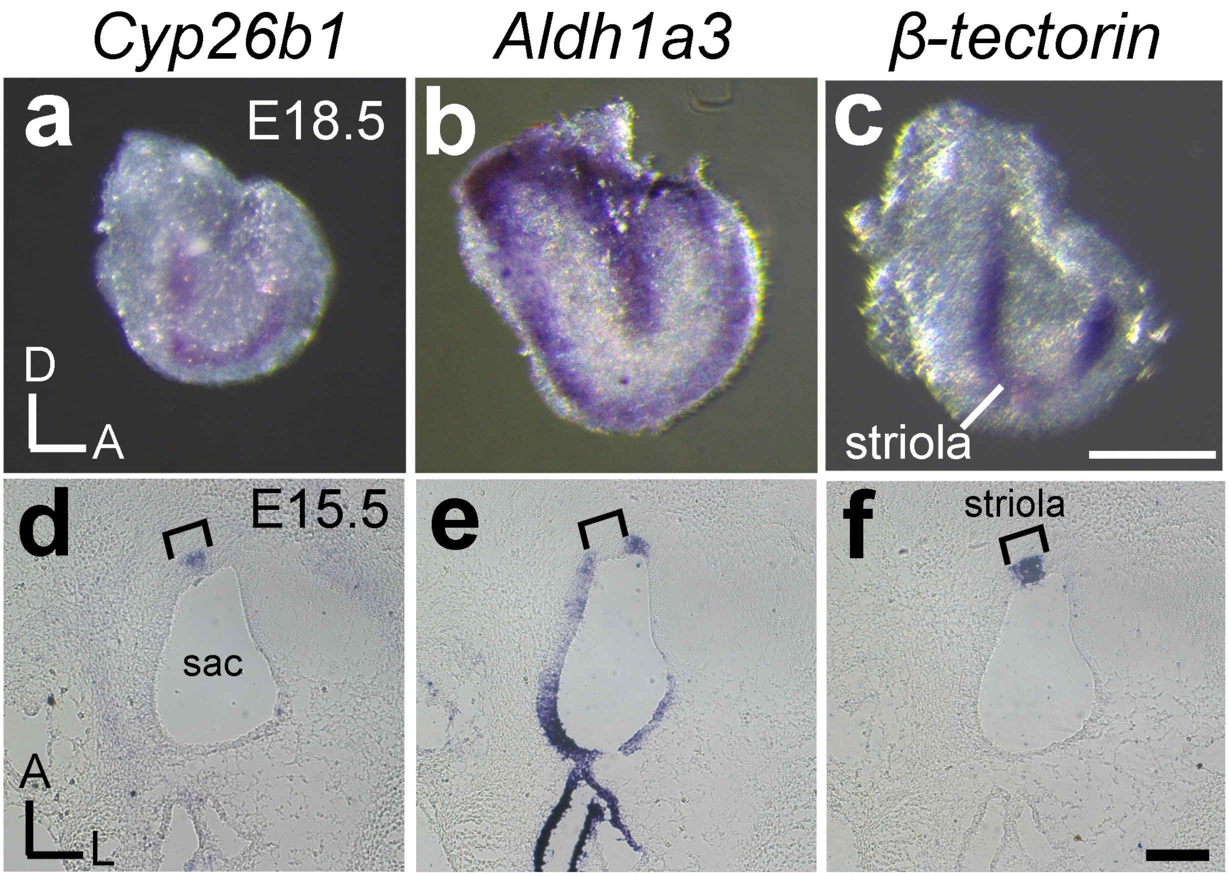

Supplementary Figure 1. Complementary expression patterns of *Cyp26b1* and *Aldh1a3* in the saccule.

(**a**-**c**) Whole mount *in situ* hybridization analyses of *Cyp26b1*, *Aldh1a3*, and *β-tectorin* in the saccule (sac) at E18.5. *Cyp26b1*-positive region corresponds to the *β-tectorin*-positive striolar region. *Aldh1a3* is predominantly expressed in the peripheral region. (**d**-**f**) Adjacent sections of E15.5 saccule showing complementary expression pattern of *Cyp26b1* and *Aldh1a3*. Scale bars for both whole mount and section images are 200 μm. A, anterior; L, lateral; D, dorsal.

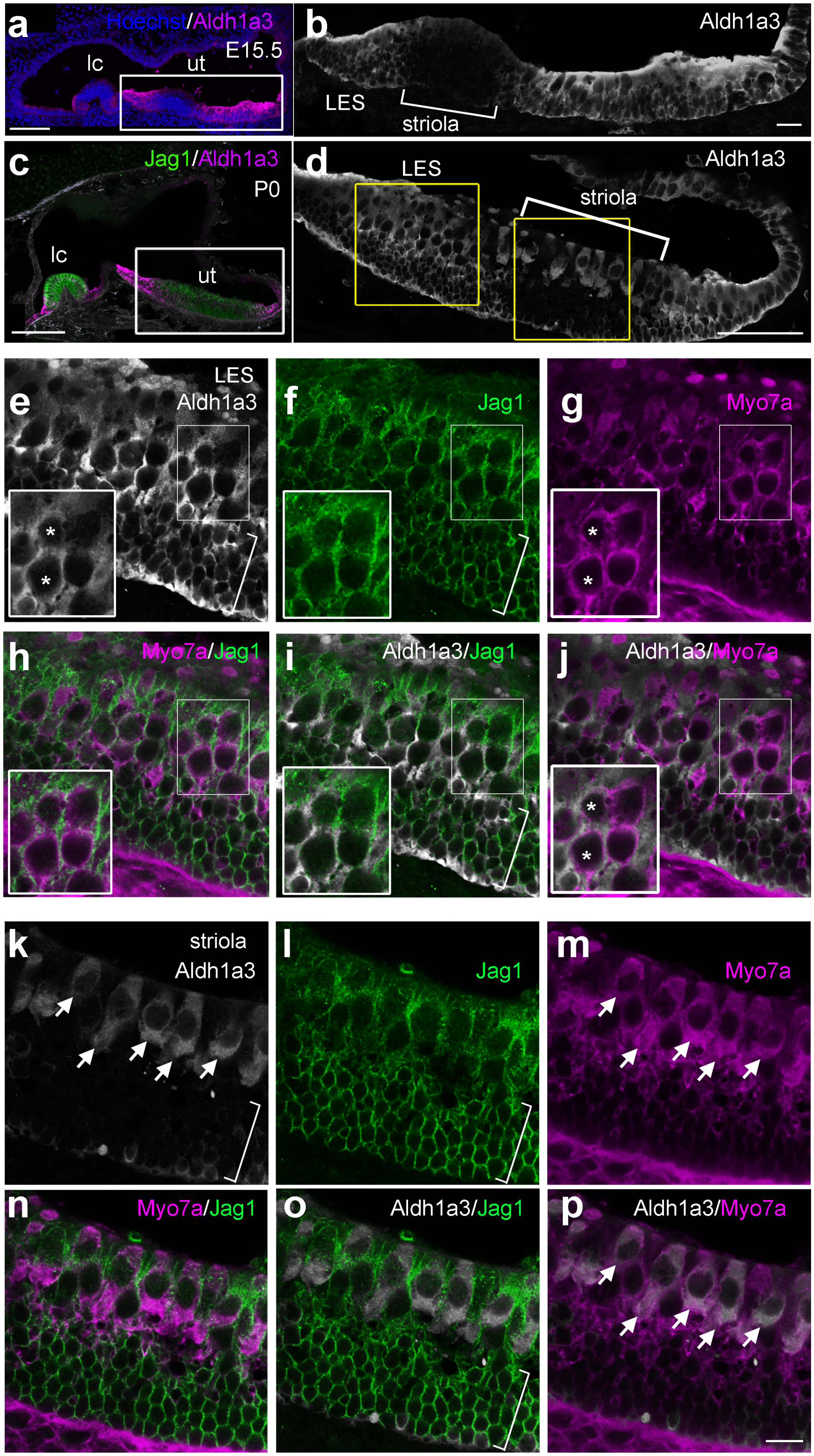
a
Hoechst/Aldh1a3
E15.5
lc
ut
b
Aldh1a3
LES
striola
c
Jag1/Aldh1a3
P0
lc
ut
d
LES
Aldh1a3
striola
e
LES
Aldh1a3
f
Jag1
g
Myo7a
h
Myo7a/Jag1
i
Aldh1a3/Jag1
j
Aldh1a3/Myo7a
k
striola
Aldh1a3
l
Jag1
m
Myo7a
n
Myo7a/Jag1
o
Aldh1a3/Jag1
p
Aldh1a3/Myo7a

Supplementary Figure 2. Anti-Aldh1a3 staining in the utricle and crista.

An E15.5 (**a**, **b**) and P0 section (**c**-**p**) of the utricle (ut) and lateral crista (lc) labeled with antibodies against Aldh1a3 only (**a**, **b**) or Aldh1a3, Myosin7a, and Jag1(**c**-**p**). (**b**) A magnified image of inset in (**a**). Consistent with the *in situ* hybridization results (Fig. 1**g**), Aldh1a3 immunoreactivity is localized to the extrastriola/peripheral zone of the utricle and lateral crista at E15.5. (**d**) A magnified image of inset in (**c**). (**e**-**j**) Magnified images of the LES in the left inset of (**d**). Aldh1a3 immunoreactivity (**e**) is localized in the Jag1-positive SC region (**e**, **f**, **i**, bracket) and in some of the Myosin 7a-positive HCs (magenta) that are surrounded by SC processes (**e**, **g**, **j**, asterisks in inset). (**k**-**p**) Magnified images of the striola in the right inset of (**d**). Aldh1a3 immunoreactivity (**k**) co-localizes with some of the Myosin 7a-positive HCs (**k**, **m**, **p**, arrows) but does not appear to be present in the Jag1-positive SCs (**k**, **l**, **o,** bracket). Scale bars; 100 μm in **a,** 20 μm in **b,** 200 μm in **c**, 50 μm in **d**, and 10 μm in **p,** which applies to **e-o.**

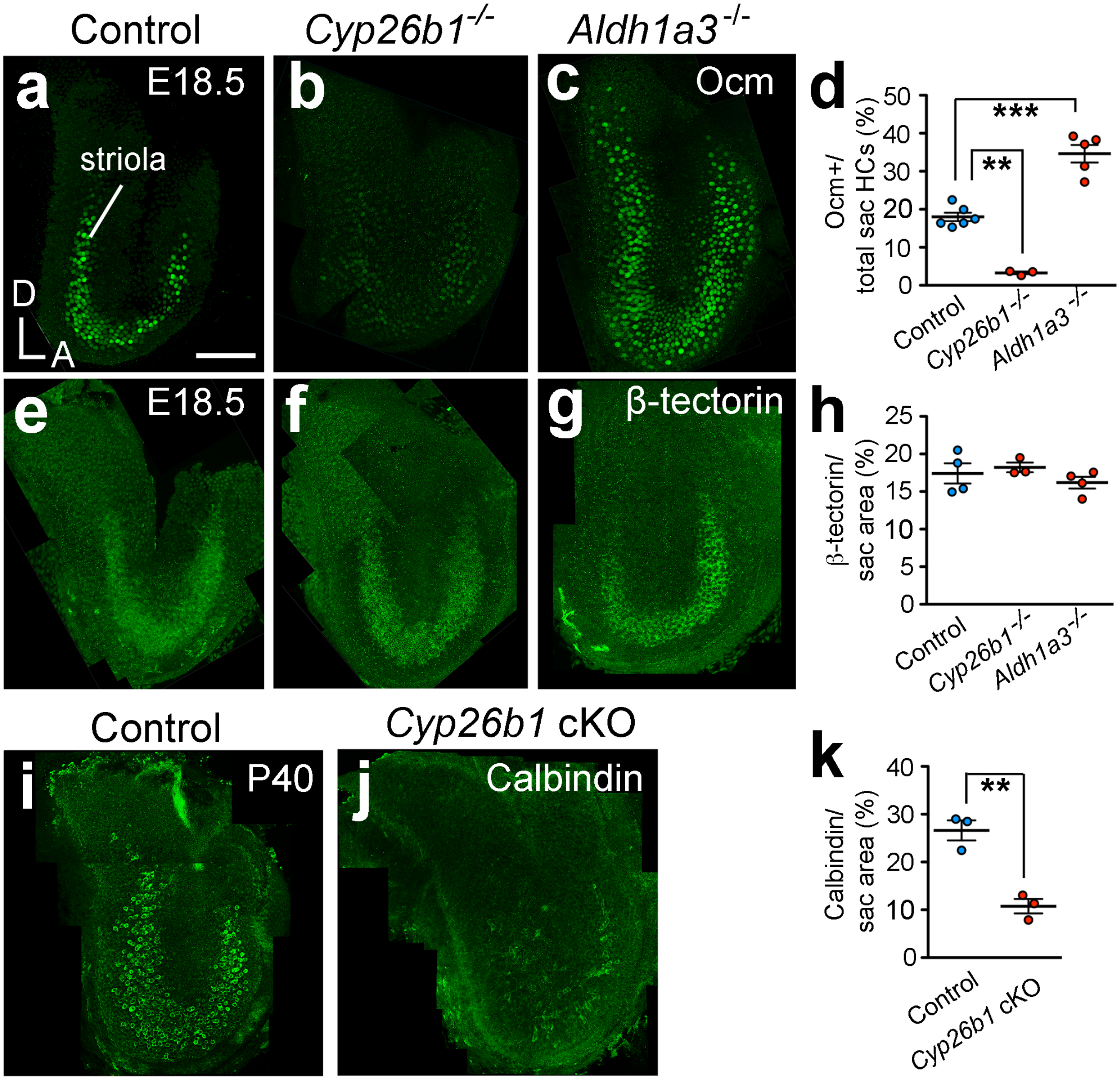

Supplementary Figure 3. Disruption of RA signaling affects striolar formation in the saccule.

(**a**-**d**) Immunohistochemistry and quantification of oncomodulin (Ocm)$^+$ HCs in E18.5 saccules (sac). Ocm$^+$ striolar type I HCs is reduced in the *Cyp26b1*$^{-/-}$ saccule (**b**, **d**, 3.1 ± 0.7 %, n = 3, P = 0.0003, one way ANOVA with tucky test) but increased in the *Aldh1a3*$^{-/-}$ saccule (**c**, **d**, 34.6 ± 2.3 %, n = 5, P < 0.0001), compared to control saccule

(**a**, **d**, 17.7 ± 2.6 % in controls, n = 6) (**e**-**h**) Immunohistochemistry and quantification of β-tectorin$^+$ SCs in E18.5 saccules. β-tectorin$^+$ SC area is not changed in either the *Cyp26b1*$^{-/-}$ (**f**, **h**, 18.5 ± 0.8 %, n = 3, P = 0.8464, one way ANOVA with tucky test) or in the *Aldh1a3*$^{-/-}$ saccule (**g**, **h**, 16.2 ± 0.8 %, n = 4, P = 0.6655), compared to control saccule (**e**, **h**, 17.4 ± 1.3 % in controls, n = 4). (**i**-**k**) Immunostaining and quantification of calbindin$^+$ afferent neurons in P40 saccules. Calbindin expression detected in striolar region of control saccules (**i**, **k**, 26.6 ± 2.1 % in controls, n = 3) is reduced in *Cyp26b1* cKO saccules (**j**, **k**, 12.1 ± 0.7 % in controls, n = 3, P = 0.0036, unpaired t-test). Error bars: SEM. ***P* < 0.01 and ****P* < 0.001. Scale bars; 200 μm. D, dorsal; A, anterior.

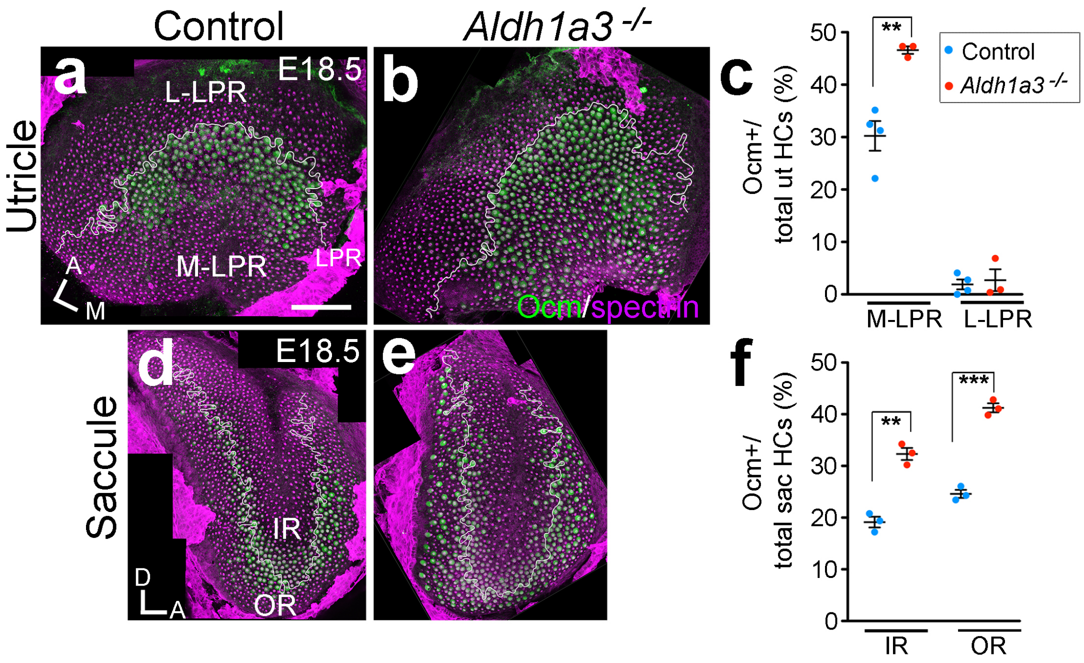

Supplementary Figure 4. Differential response to loss of *Aldh1a3* in the utricles and saccules.

(**a**-**f**) Immunohistochemistry and quantification of Ocm^{+} HCs in E18.5 utricles (ut) and saccules (sac). (**a**, **c**) Ocm, which labels striolar type I HCs in the control utricles, is only expressed medial (M-LPR, 30.2 ± 2.8%, n = 4) but not lateral (L-LPR, 1.9 ± 0.9%) to the line of polarity reversal (LPR). (**b**, **c**) Ocm staining in *Aldh1a3*$^{-/-}$ utricles is increased in the medial (47.3 ± 2.8%, n = 3, P = 0.0048, unpaired t-test) but not the lateral region (0.6 ± 0.2%, P = 0.7101, unpaired t-test). (**d**, **f**) In control saccule, striola straddles the LPR with Ocm^{+} HCs being expressed in inner (IR, 20.1 ± 0.6%, n = 3) and outer (OR, 25.2 ± 0.7%) regions of the organ. (**e**, **f**) In contrast to the utricle, Ocm-positive region is expanded on both IR (33.4 ± 0.7%, n = 3, P = 0.0011, unpaired t-test) and OR (40.4 ± 0.5%, P = 0.0001, unpaired t-test) of the LPR in *Aldh1a3*$^{-/-}$ saccules. Error bars: SEM. ***P* < 0.01 and ****P* < 0.001. Scale bar; 200 μm. A, anterior; M, medial; D, dorsal.

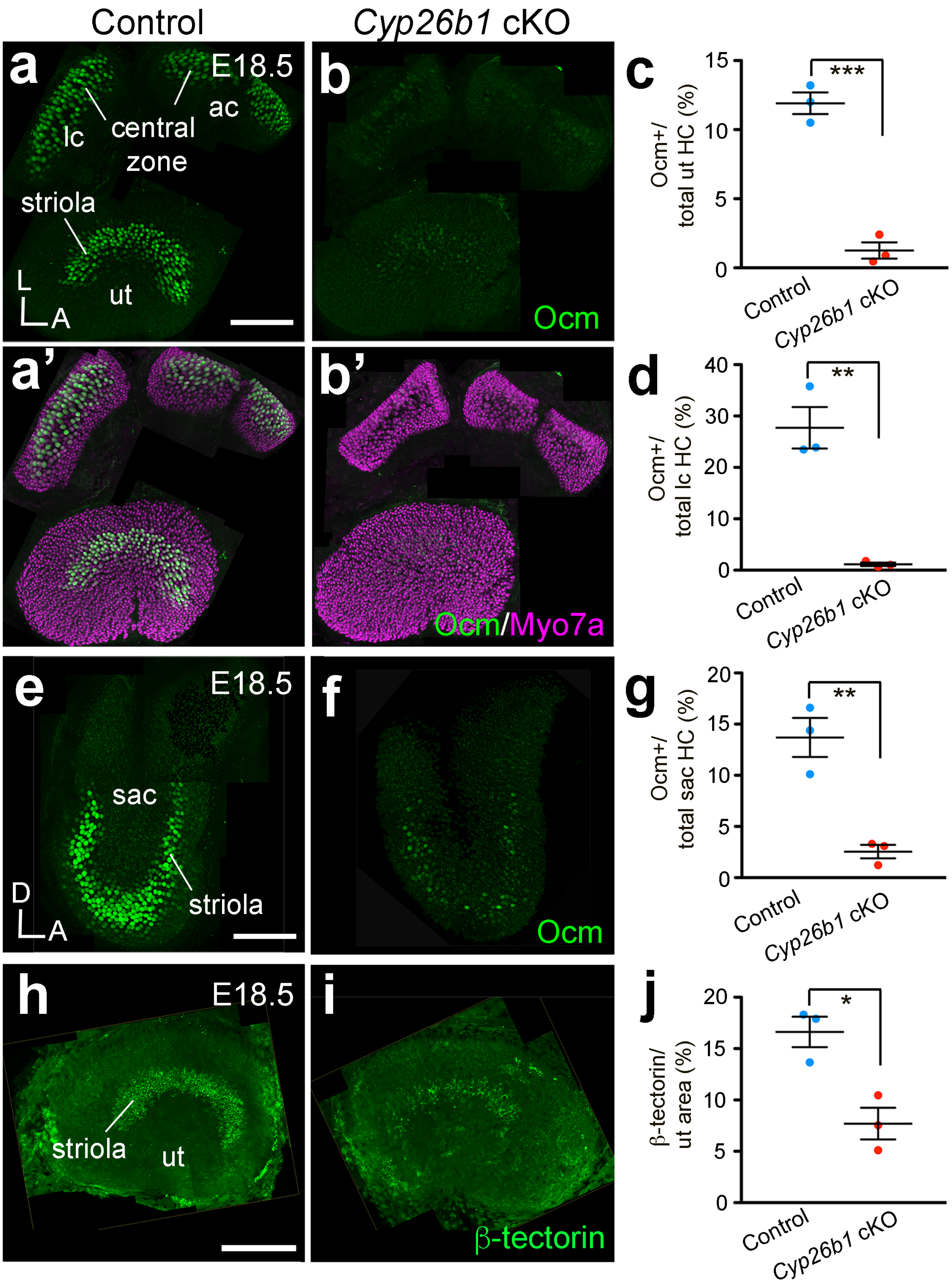

Control
Cyp26b1 cKO
a
E18.5
lc
central
zone
ac
striola
ut
L
A
b
Ocm
c
15
10
5
0
***
Ocm+/
total ut HC (%)
Control
Cyp26b1 cKO
a'
b'
Ocm/Myo7a
d
40
30
20
10
0
**
Ocm+/
total lc HC (%)
Control
Cyp26b1 cKO
e
E18.5
sac
striola
D
A
f
Ocm
g
20
15
10
5
0
**
Ocm+/
total sac HC (%)
Control
Cyp26b1 cKO
h
E18.5
striola
ut
i
β-tectorin
j
20
15
10
5
0
*
β-tectorin/
ut area (%)
Control
Cyp26b1 cKO

Supplementary Figure 5. Formation of the striolar/central zone is compromized in vestibular organs of *Cyp26b1* cKO.

Immunostaining of HCs in the utricle (ut), anterior crista (ac), and lateral crista (lc, **a**-**b'**) or the saccule (**e**-**f**) with anti-myosin7a (magenta) and anti-oncomodulin (Ocm; green) antibodies at E18.5. (**a**, **a'**) In *Foxg1*^{Cre}*;Cyp26b1*$^{lox/+}$ controls, Ocm is expressed in type I HCs of striola and central zones, whereas all HCs are positive for Myosin7a. (**b**-**b'**) Ocm expression is reduced in *Cyp26b1* cKO utricles (**b**-**c**, 1.2 ± 0.6 %, n = 3, P = 0.0004, unpaired t-test), lateral cristae (**b**-**d**, 1.2 ± 0.3 %, n = 3, P = 0.0028), and saccules (**f**-**g**, 2.2 ± 0.9 %, n = 3, P = 0.0052) when compared to control utricles (12.0 ± 0.8 % in controls, n = 3), lateral cristae (27.7 ± 4.0 %, n = 3), and saccules (13.3 ± 3.2 %, n = 3), respectively. (**h**-**j**) β-tectorin immunoreactivity that only labels striolar SCs of control utricles (**h**, **j**, 16.6 ± 1.4 % in controls, n = 3) is reduced in *Cyp26b1* cKO utricles (**i**-**j**, 7.6 ± 1.5 %, n = 3, P = 0.0141, unpaired t-test). Error bars: SEM. A, anterior; L, lateral; D, dorsal. *P < 0.05, **P < 0.01, and ***P < 0.001. Scale bars; 200 μm.

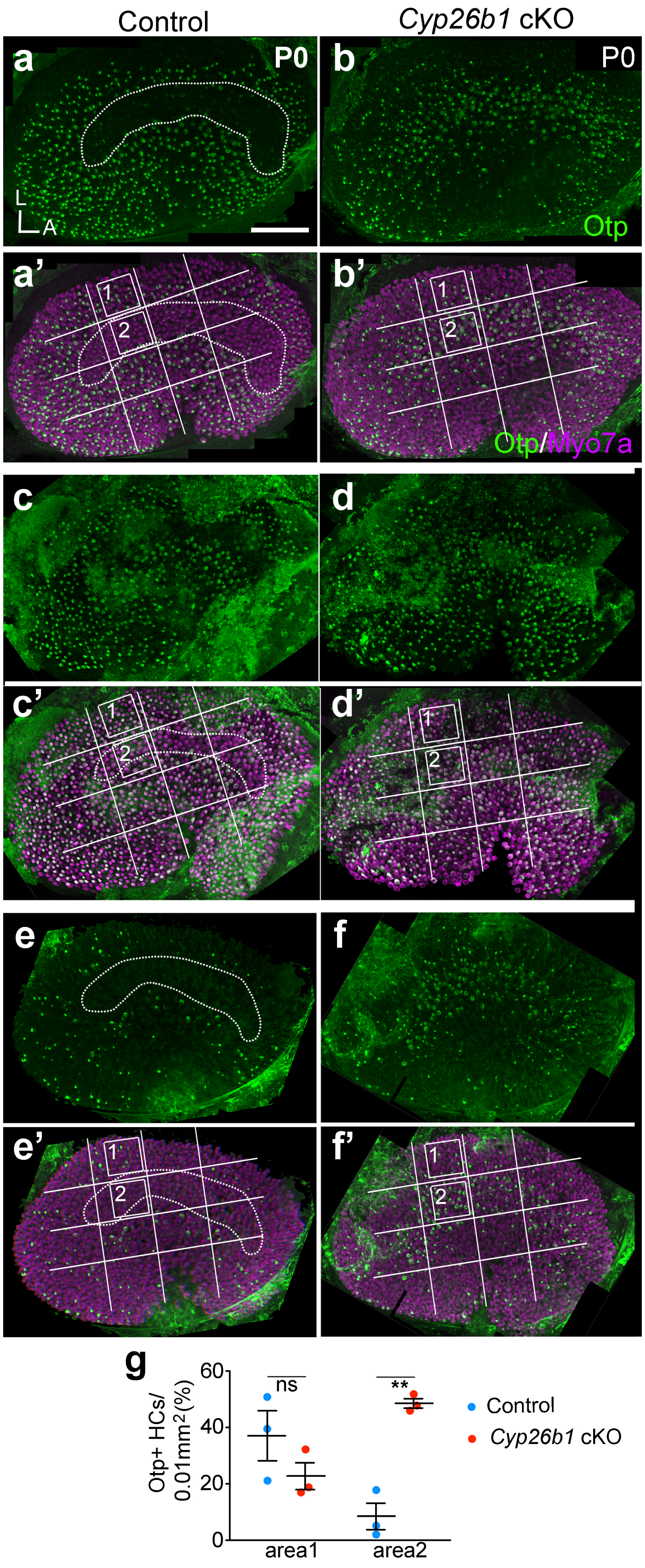

Control
Cyp26b1 cKO
a
P0
b
P0
L
A
Otp
a'
b'
1
2
Otp/Myo7a
c
d
c'
d'
e
f
e'
f'
g
Otp+ HCs/ 0.01mm2(%)
60
40
20
0
ns
**
area1
area2
Control
Cyp26b1 cKO

Supplementary Figure 6. Increased osteopontin immunoreactivity in utricles of *Cyp26b1* cKO mice.

(**a**-**g**) Immunohistochemistry and quantification of osteopontin$^+$ HCs (Otp) in P0 utricles. Areas 1 and 2 correspond to the LES and striola, respectively (see Methods). (**a**, **b**), (**c**, **d**), (**e**, **f**) are littermates of control (**a**, **c**, **e**) and *Cyp26b1* cKO (b, d, f) utricles. At P0, Otp-positive staining in type I extrastriolar HCs (green) is emerging in the extrastriola (**a**-**a'**, **c**-**c'**, **e**-**e'**, **g**, 37.1 ± 8.8 % Otp+/Myo7a HC in area 1 of LES, n = 3) but the striola shows fewer Otp-positive HCs (**a**-**a'**, **c**-**c'**, **e**-**e'**, **g**, 8.4 ± 4.7 % in area 2 of striola, n = 3). In contrast, percentages of Otp-positive HCs in *Cyp26b1* cKO utricles are increased in area 2 compared to controls (**b**-**b'**, **d**-**d'**, **f**-**f'**, **g**, 48.5 ± 1.6 %, n = 3, P = 0.0013, unpaired t-test). No difference in percentages of Otp-positive HCs are observed between control and mutant utricles in LES (**b**-**b'**, **d**-**d'**, **f**-**f'**, **g**, 22.7 ± 4.8 % in area 1, n = 3, P = 0.2268, unpaired t-test). Error bars: SEM. Scale bar; 200 μm. A, anterior; L, lateral. ***P* < 0.01.

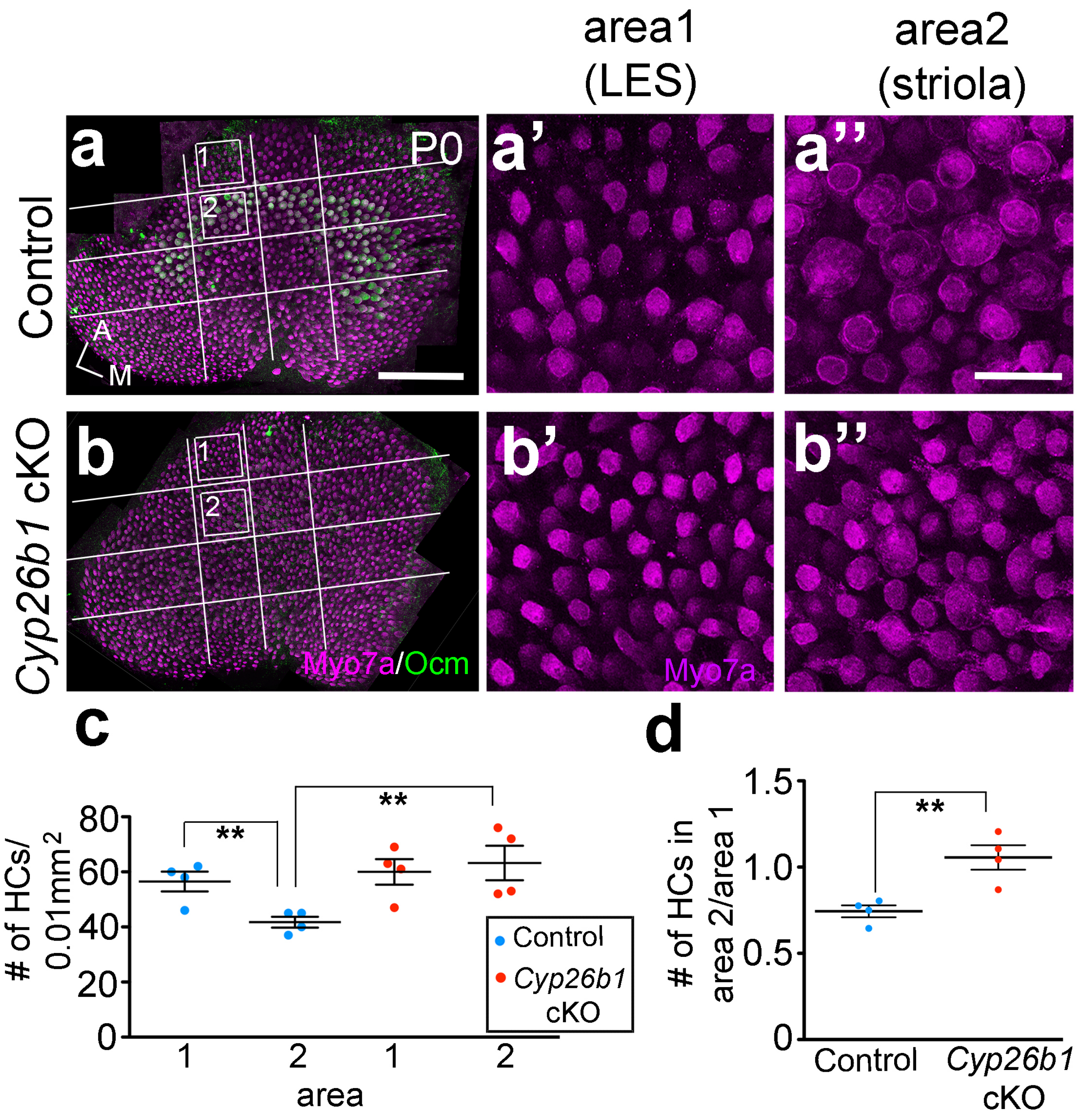

Supplementary Figure 7. Loss of regional difference in HC density in *Cyp26b1* cKO utricles.

(**a**-**c**) Immunohistochemistry and quantification of Myosin7a^{+} (magenta) HCs in P0 utricles. Ocm (green) marks control striola. The posterior half of the middle-third region of each utricle is sub-divided into four regions (see Methods for details). In area 1 of control utricles, which represents the lateral extrastriola (LES), HCs are smaller and

densely packed (**a'**, **c**, 56.5 ± 3.5 per 0.01 mm^2, n = 4). HCs are larger and wider apart in area 2, which represents striolar region in controls (**a''**, **c**, 41.7 ± 1.9 per 0.01 mm^2, P = 0.0057, two-way ANOVA with multiple comparisons). In *Cyp26b1* cKO mutant utricles, area 2 have smaller and denser HCs (**b''**, **c**, 63.2 ± 6.2 per 0.01 mm^2, n = 4,), similar to HCs in area1 (**b'**, **c**, 60.0 ± 4.6 per 0.01 mm^2, P = 0.3457, two-way ANOVA with multiple comparisons). (**d**) Averaged ratio of HC density in area 2 to area 1 in control (0.74 ± 0.03), is smaller than *Cyp26b1* cKO mutant utricles (1.05 ± 0.07, P = 0.0037, unpaired t-test). a, anterior; m, medial. Error bars: SEM. A, anterior; M, medial. **P < 0.01. Scale bar; 200 μm for (**a**, **b**), and 30 μm (**a'**, **a"**, **b'**, **b"**).

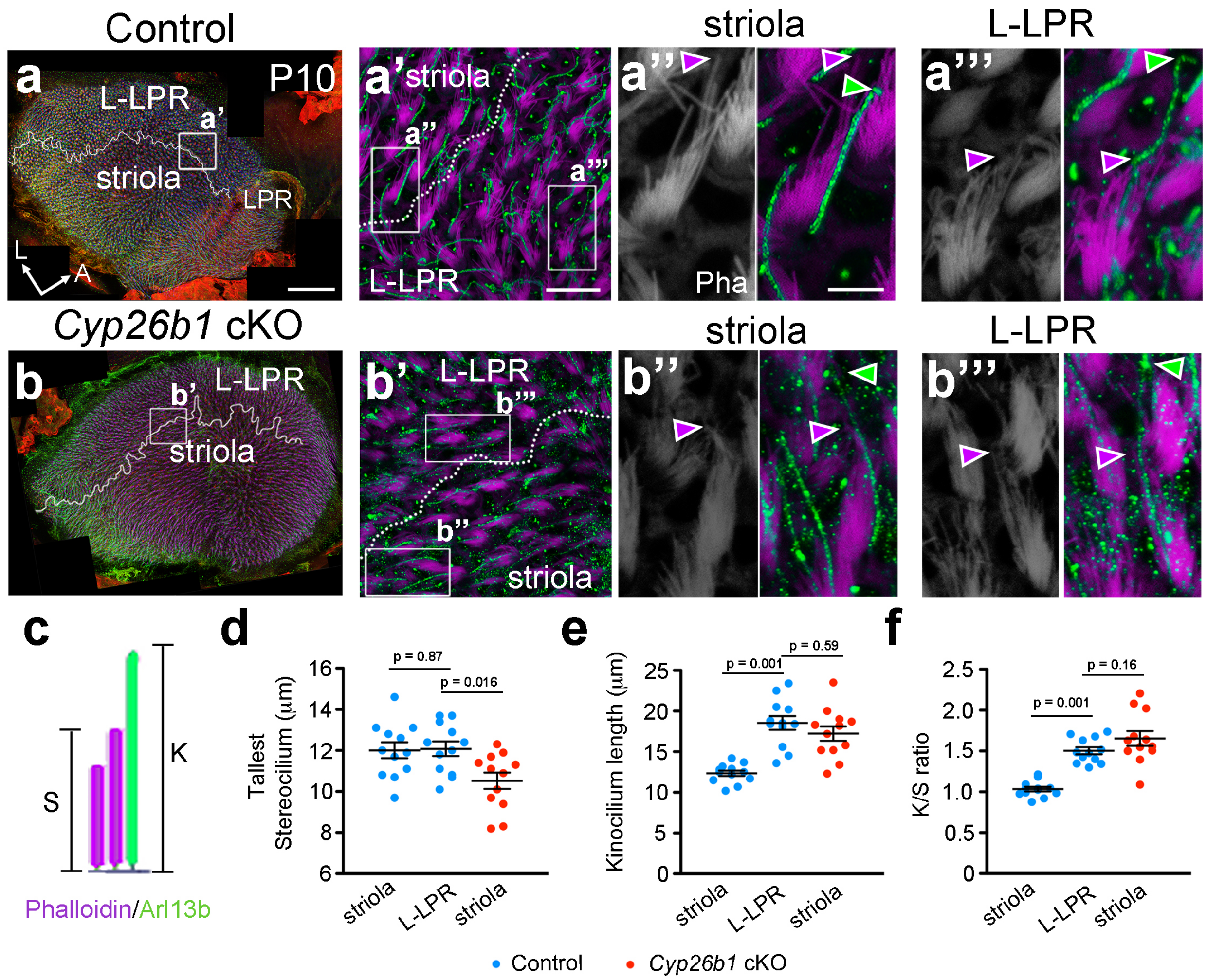

Supplementary Figure 8. Loss of regional differences in kinocilium length and K/S ratio of hair bundles in *Cyp26b1* cKO utricles.

(**a**-**f**) Immunohistochemistry and measurements of cilia length and the ratio of lengths of the kinocilium to tallest stereocilia (K/S ratio) in P3-10 utricles of control and *Cyp26b1* cKO mutant ears. The utricles were labeled with phalloidin (magenta, labeling stereocilia) and antibodies to Arl13b (green, specific for labeling the kinocilium). The LPR was used to approximate the putative striolar region. In the striola of control

utricles (**a’**, clockwise rotation of the inset in **a**), the height of the kinocilium and the tallest stereocilium of a hair bundle is similar to each other (**a’’**, **c**-**f**, kinocilium length: 12.1 ± 0.3 μm, tallest stereocilum length: 11.8 ± 0.3 μm, K/S ratio: 1.03 ± 0.02, n = 12/4, 12 hair bundles measured from 4 specimens), whereas the kinocilium is longer than the tallest stereocilium in HCs of L-LPR (**a’’’**, **c**-**f**, kinocilium length: 18.2 ± 0.8 μm, tallest stereocilum length: 12.0 ± 0.3 μm, K/S ratio: 1.50 ± 0.04, n = 12/4). In contrast, kinocilium lengths of HCs in the striolar region of mutant utricles (**b’**) are longer than the tallest stereocilium (**b’’**, **c**-**f**, kinocilium length: 17.3 ± 0.7 μm, tallest stereocilum length: 10.6 ± 0.3 μm, K/S ratio: 1.64 ± 0.07, n = 12/4), which are comparable to those in L-LPR (**a’’’**, **b’’’**). Green and magenta arrowheads indicate the approximate tips of kinocilium and the tallest stereocilium, respectively. The one-way ANOVA with multiple comparisons was applied. Error bars: SEM. L, lateral; A, anterior. Scale bar; 200 μm for **a**, **b**; 30 μm for **a’ b’**; and 10 μm for **a’’**, **b’’**.

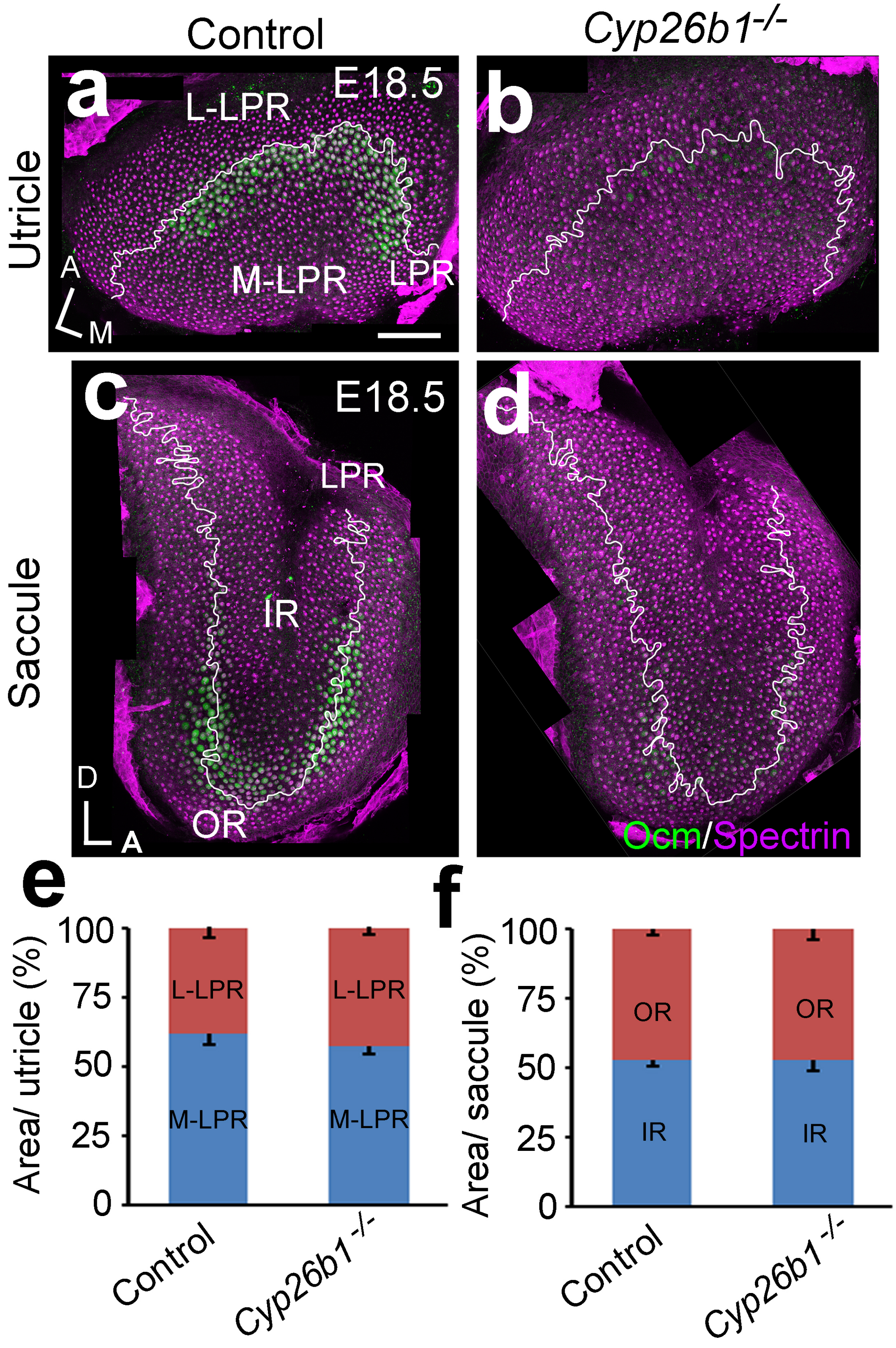
Control
Cyp26b1-/-
Utricle
Saccule
a
E18.5
L-LPR
M-LPR
LPR
A
M
b
c
E18.5
LPR
IR
OR
D
A
d
Ocm/Spectrin
e
Area/ utricle (%)
100
75
50
25
0
L-LPR
M-LPR
L-LPR
M-LPR
Control
Cyp26b1 -/-
f
Area/ saccule (%)
100
75
50
25
0
OR
IR
OR
IR
Control
Cyp26b1 -/-

Supplementary Figure 9. Position of the line of polarity reversal (LPR) is maintained in *Cyp26b1*$^{-/-}$ utricles and saccules.

(**a**-**f**) E18.5 of controls and *Cyp26b1*$^{-/-}$ utricles and saccules that were stained with antibodies against Ocm (green) and Spectrin (magenta), which labels the cuticular plate of HCs and identifies the hair bundle orientation[1]. In control utricles, the LPR (white line in **a**) is located lateral to the Ocm^{+} striola, whereas it bisects the striola in the saccule (white line in **c**). The LPR is located approximately one-third into the utricle from the lateral edge (61.6 ± 2.1% M-LPR, n = 3) and halve area of the saccule (52.6 ± 1.3% IR, n = 3). In *Cyp26b1* mutants, the position of the LPR remains in its relative position in both utricle (57.1± 1.6% M-LPR, n = 3, P = 0.1609, unpaired t-test) and saccule (52.8 ± 2.6% IR, n = 3, P = 0.9600, unpaired t-test) despite the loss of Ocm immunostaining. Error bars: SEM. A, anterior; M, medial; D, dorsal. Scale bar, 200 μm.

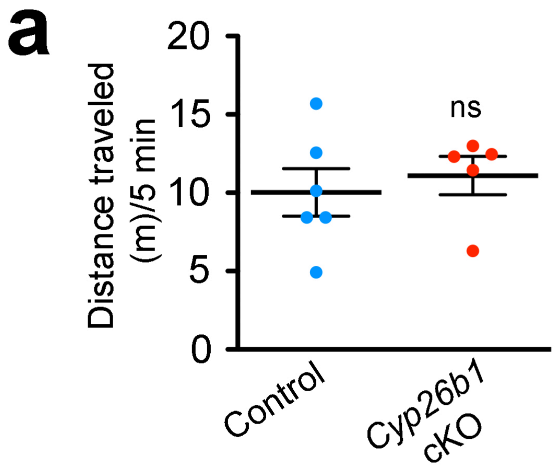

Supplementary Figure 10. No hyperactivity in *Cyp26b1* cKO mice.

(**a**) Quantification of open field tests for control and mutant mice. Measurements of total distance a mouse traveled over a 5-minute period when placed in the corner of an open field box. No difference between controls and mutants was observed (P = 0.6072, unpaired t-test). Error bars: SEM.

Supplementary Table

Table1: Primers for genotyping *Aldh1a3*$^{-/-}$, *Cyp26b1*$^{-/-}$ and *Cyp26b1*^{lox} mice[2,3].

Aldh1a3 Fw	5′-GCCATAAAAGCTGGGGTGTCTG-3′
Aldh1a3 Rv	5′-TGGATGGATGGATGGGTGATG-3′
Cyp26b1 P1	5'-AAGTACACCTGGCAGACATG-3'
Cyp26b1 P2	5'-CCTGTCCCATATTTATTCACTGAC-3'
Cyp26b1 P3	5'-CTCCTCTTAAAGCTTCTCTA-3'

Supplementary References

1 Deans, M. R. *et al.* Asymmetric distribution of prickle-like 2 reveals an early underlying polarization of vestibular sensory epithelia in the inner ear. *J Neurosci* **27**, 3139-3147, doi:10.1523/JNEUROSCI.5151-06.2007 (2007).

2 Okano, J. *et al.* Increased retinoic acid levels through ablation of Cyp26b1 determine the processes of embryonic skin barrier formation and peridermal development. *J Cell Sci* **125**, 1827-1836, doi:10.1242/jcs.101550 (2012).

3 Molotkov, A., Molotkova, N. & Duester, G. Retinoic acid guides eye morphogenetic movements via paracrine signaling but is unnecessary for retinal dorsoventral patterning. *Development* **133**, 1901-1910, doi:10.1242/dev.02328 (2006).
