# Supplementary material for: Retinoic acid degradation shapes zonal development of vestibular organs and sensitivity to transient linear accelerations
Source: Nat Commun. 2020 Jan 2;11:63. doi: 10.1038/s41467-019-13710-4 (PMC6940366; doi:10.1038/s41467-019-13710-4)
Supplement: Supplementary file 3 — Reporting Summary [file 41467_2019_13710_MOESM3_ESM.pdf]

nature research

Corresponding author(s): Doris Wu

Last updated by author(s): Oct 22, 2019

Reporting Summary

Nature Research wishes to improve the reproducibility of the work that we publish. This form provides structure for consistency and transparency in reporting. For further information on Nature Research policies, see Authors & Referees and the Editorial Policy Checklist .

Statistics

For all statistical analyses, confirm that the following items are present in the figure legend, table legend, main text, or Methods section.

n/a	Confirmed	
☐	☒	The exact sample size (n) for each experimental group/condition, given as a discrete number and unit of measurement
☐	☒	A statement on whether measurements were taken from distinct samples or whether the same sample was measured repeatedly
☐	☒	The statistical test(s) used AND whether they are one- or two-sided<br>*Only common tests should be described solely by name; describe more complex techniques in the Methods section.*
☒	☐	A description of all covariates tested
☐	☒	A description of any assumptions or corrections, such as tests of normality and adjustment for multiple comparisons
☐	☒	A full description of the statistical parameters including central tendency (e.g. means) or other basic estimates (e.g. regression coefficient) AND variation (e.g. standard deviation) or associated estimates of uncertainty (e.g. confidence intervals)
☐	☒	For null hypothesis testing, the test statistic (e.g. F, t, r) with confidence intervals, effect sizes, degrees of freedom and P value noted<br>*Give P values as exact values whenever suitable.*
☒	☐	For Bayesian analysis, information on the choice of priors and Markov chain Monte Carlo settings
☒	☐	For hierarchical and complex designs, identification of the appropriate level for tests and full reporting of outcomes
☒	☐	Estimates of effect sizes (e.g. Cohen's d, Pearson's r), indicating how they were calculated

Our web collection on statistics for biologists contains articles on many of the points above.

Software and code

Policy information about availability of computer code

Data collection	Scanning confocal microscope: LSM780, Upright microscope: ZEN (2012), Patch clump: Patchmaster and JPCalc (11), VsEP (BioSig), aVOR: LabVIEW, OVAR: iScan, Rotarod: ROTAROD, Scanning electron microscopy: SU4800, Head movement: CoolTerm
Data analysis	ImageJ (1.52h), Prism (7), OriginPro (2017), Topscan (3.0), MATLAB

For manuscripts utilizing custom algorithms or software that are central to the research but not yet described in published literature, software must be made available to editors/reviewers. We strongly encourage code deposition in a community repository (e.g. GitHub). See the Nature Research guidelines for submitting code & software for further information.

Data

Policy information about availability of data

All manuscripts must include a data availability statement. This statement should provide the following information, where applicable:

- Accession codes, unique identifiers, or web links for publicly available datasets
- A list of figures that have associated raw data
- A description of any restrictions on data availability

The datasets generated during and/or analysed during the current study are available from the corresponding author on reasonable request.

Field-specific reporting

Please select the one below that is the best fit for your research. If you are not sure, read the appropriate sections before making your selection.

☒ Life sciences ☐ Behavioural & social sciences ☐ Ecological, evolutionary & environmental sciences

October 2018

Life sciences study design

All studies must disclose on these points even when the disclosure is negative.

Sample size	Sample size was described in Methods section, the main text or figure legend, if not already plotted on individual graphs in the figures. All quantifications are results from at least three samples.
Data exclusions	No data were excluded from the analysis.
Replication	Replications are indicated in the Methods section and figure legend of each graph. All in situ hybridization results were reproducible and conducted at least twice.
Randomization	Sample collection is randomized based on the litters collected as they become available.
Blinding	During data collection, investigators were not blinded. However, data collection and analyses of behavioral tests of mice were blinded.

Reporting for specific materials, systems and methods

We require information from authors about some types of materials, experimental systems and methods used in many studies. Here, indicate whether each material, system or method listed is relevant to your study. If you are not sure if a list item applies to your research, read the appropriate section before selecting a response.

Materials & experimental systems

n/a	Involved in the study	
☐	☒	Antibodies
☒	☐	Eukaryotic cell lines
☒	☐	Palaeontology
☐	☒	Animals and other organisms
☒	☐	Human research participants
☒	☐	Clinical data

Methods

n/a	Involved in the study	
☒	☐	ChIP-seq
☒	☐	Flow cytometry
☒	☐	MRI-based neuroimaging

Antibodies

Antibodies used	goat polyclonal anti-oncomodulin (1:300, Santa Cruz Biotech, #sc-7446), rabbit polyclonal anti-b-tectorin (1:1000, a gift from Guy Richardson, U of Sussex), rabbit polyclonal anti-Myosin7a (1:1000, Proteus, #25-6790), mouse monoclonal anti-Myosin7a (1:50, Santa Cruz, sc-74516), rabbit polyclonal anti-Calbindin (1:1000, Millipore, #AB1778), mouse monoclonal anti-bIII tubulin (1:500; R&D, #MAB1195), mouse anti-βII-spectrin (1:500; BD Bioscience, #612562), rabbit polyclonal anti-aII spectrin (1:500; Invitrogen, #PA5-35383), goat polyclonal anti-Jag1 (1:200; Santa Cruz, #sc-6011), goat polyclonal anti-Sox2 (1:500; Santa Cruz Biotech, #sc-17320), mouse monoclonal anti-Sox2 (1:150; Santa Cruz, sc-365823), goat polyclonal anti-osteopontin (1:500; R&D systems, #AF808), rabbit polyclonal anti-Aldh1a3 (1:150; Millipore, #ABN427), and mouse monoclonal anti-acetylated Tubulin (1:1000; Sigma-Aldrich, #T6793).
Validation	All antibodies used are known to work well on mouse tissue.

Animals and other organisms

Policy information about studies involving animals; ARRIVE guidelines recommended for reporting animal research

Laboratory animals	The following mouse strains were used in the study: Aldh1a3+/- (maintained in a mixed C57BL/6J and CD1 background)1, Cyp26bflox/flox (RIKEN BRC (RBRC04333), maintained in a C57BL/6J background) and Foxg1Cre (RRID:IMSR_JAX:004337, maintained in C57BL/6J background). In addition, Cyp26b1+/- mice were generated by breeding Cyp26b1flox/flox mice with a ubiquitous cre line, ActinCre, and maintained in a C57BL/6J background after recombination and removal of the cre allele. Foxg1Cre; Cyp26b1flox/- were produced by crossing Foxg1Cre;Cyp26b1+/- males with Cyp26b1flox/flox females.
Wild animals	not used
Field-collected samples	not used
Ethics oversight	All animal experiments were conducted under the approved NIH animal protocols at the NIH, University of Chicago, University of Nebraska -Lincoln, Johns Hopkins University and according to NIH animal user guidelines.

Note that full information on the approval of the study protocol must also be provided in the manuscript.
