# Supplementary material for: Retinoic acid degradation shapes zonal development of vestibular organs and sensitivity to transient linear accelerations
Source: Nat Commun. 2020 Jan 2;11:63. doi: 10.1038/s41467-019-13710-4 (PMC6940366; doi:10.1038/s41467-019-13710-4)
Supplement: Supplementary file 4 — Description of Additional Supplementary Files [file 41467_2019_13710_MOESM4_ESM.docx]

**Description of Additional Supplementary Files**

File name: Supplementary Movie 1.
Description: Open field behavior of Cyp26b1 cKO mice. No obvious behavioral problems except slight head tremors are detectable in Cyp26b1 cKO mutants.

File name: Supplementary Movie 2.
Description: Cyp26b1 cKO mice traversing on a balance beam. Compared to controls, Cyp26b1 cKO mice are uncoordinated while traversing a 6 mm narrow beam.

File name: Supplementary Movie 3.
Description: Head tremor of Cyp26b1 cKO mice at P9. Cyp26b1 cKO mice exhibit increased head tremor during self-motion.
