# Supplementary material for: Retinoic acid degradation shapes zonal development of vestibular organs and sensitivity to transient linear accelerations
Source: Nat Commun. 2020 Jan 2;11:63. doi: 10.1038/s41467-019-13710-4 (PMC6940366; doi:10.1038/s41467-019-13710-4)
Supplement: Supplementary file 2 — Peer Review [file 41467_2019_13710_MOESM2_ESM.pdf]

Reviewers' comments:

Reviewer #1 (Remarks to the Author):

The report by Ono et al. show that formation of the striolar/central zones of the vestibular organs in mice during embryogenesis requires Cytochrme P450. In Cyp26b1 KO mice the authors describe how the distinctive identities of these zones are compromised. This includes a reduction in the numbers of Type ! hair cells within the striolar/central zones, a reduction in the number of calyx-only and complex calyx endings, and loss of size differentiation among otoconia over the strioloar zone. Physiologically, there were no differences in threshold currents between striolar and extra-striolar afferents.

Because of their unique dynamic properties, irregular afferents from the striolar region are believed to be the primary generators of the vestibular short latency evoked potential (VsEP). Consistent with the observed morphological and physiological changes, the VsEP was significantly altered or absent in the Cyp26b1 KO mice. Interestingly, these mice performed better on roto-rod tests and appeared to have relatively normal VOR and OVAR responses.

This paper reports an extensive array of experimental maneuvers focussed on the development of the striolar/central zones in mice and on vestibular behaviors that may be dependent on this specialization. The data are presented in a logical order and clearly described. The behavioral data including the VsEP measurements are relatively standardized and performed by experienced investigators. Although the number of animals used in any one test was small, the data were statistically significant and the differences between control and KO mice were large and evident.

This is a paper describes several notable findings of particular interest to vestibular scientists (e.g., the sources of the VsEP and the contribution of extra-striolar afferents to the VOR and to vestibulo-spinal reflexes) and developmental mechanisms that determine specific sensory specializations. Although the data of individual maneuvers are presented briefly and succinctly, the overall conclusion appears to be well founded and will be of broad interest to the field.

Reviewer #2 (Remarks to the Author):

It is well established that the vestibular maculae are divided into anatomically well-distinguished zones, but their relevance for specific vestibular functions is poorly understood. This study by Ono et al. makes a decisive contribution to fill this gap in knowledge. Based on overall very convincing data, the authors show that the specification of the central/striola part of the utricle depends on RA signaling. By genetically manipulating the developmental specification of this zone, they show that the central/striola zone is essential for detecting transient responses in linear acceleration. The conclusions are supported by data of excellent quality, with the exception of a few points that will be outlined below.

It is well known that RA gradients, established by RA producing sources and metabolizing sinks, are important for setting up certain axis during organ development, and a previous study (Romand et al) demonstrated that Aldh1a3 (a RA source) and Cyp26b1 (a sink) exhibit complementary expression patterns in the cochlea. The findings presented in this paper therefore are not entirely surprising or conceptually new. However, this study's genetic deletion of Cyp26b1 produces a strikingly specific and clean phenotype (loss of striola specification), which allowed the authors to definitively dissect the functional role of the utricular striola. The complete loss of VsEP in a mouse that is normal in most other vestibular functions is a powerful phenotype, and provides a useful basis to dissect out the different vestibular modalities.

What prevents this study from being complete is the lack of any effort to dissect the mechanism by which RA signaling leads to the zonal specification, including the cellular phenotypes in SC and HCs. This reviewer however thinks that this manuscript has sufficient novelty, significance and substance to justify publication in a high-profile journal, without delineating a clear mechanistic understanding,

provided the following concerns can be addressed in a satisfactory manner:

- Figure 1: the expression pattern of Aldh1a3, Cyp26b1 and β-tectorin mRNA in the vestibular macula is convincing and consistent with previous reports in the cochlea, and the quality of the in situs is appropriate. It would be better however to show the protein expression pattern. The authors must have tried immunofluorescence. Did the IFs not work? IFs would allow a more convincing confirmation that Aldh1a3 and Cyp26b1 are indeed expressed in SCs.
- When the Aldh1a3 and Cyp26b1 constitutive KO mice are introduced the first time, it would be helpful to mention that these mice die around birth, but are ok for embryonic studies. This also sets up the rationale for the conditional KOs.
- Line 156 states that there is no apparent loss of total number of MYO7A positive hair cells, without providing any data. This needs to be quantified, especially since later in the manuscript, it is reported that the density of hair cells changes in the striolar region of the conditional Cyp26b1 mice (line 232).
- Line 194: The authors state: "In Cyp26b1 cKO mice, Ocm and β-tectorin phenotypes are similar to those of the Cyp26b1 knockouts (data not shown)." Data not shown is not acceptable in this case. The authors need to provide data for this.
- Starting line 238, the authors investigate morphological phenotypes in hair cells in the Cyp26b1 mutant versus the WT mice:
a) it would be good to learn whether the number or ratio of type I and type II hair cells changes in the mutant mice. This has not been investigated in this study.
b) the immunofluorescence images used to quantify the kinocilium/stereocilia length ratio are of insufficient quality. Please provide better IF images, and use imaging tools such as Imaris to render 3D stacks and determine lengths of stereocilia and kinocilia in 3D space. Or better, conduct analysis on SEMs.
c) in addition to the relative ratio, the absolute length is here of interest. Especially since a failed coupling of the kinocilia/stereocilia with the gelatinous zone/otoconia might cause the functional defects.

Discussion:
- it seems the RALDH3 mouse phenotype differs from a previous study. Any idea why that would be the case?

In summary, this is a very nice study that makes a significant contribution to our understanding of vestibular sensation. It is of general interest for the wider Neuroscience community since it provides the cellular basis for a sensory submodality. The lack of mechanistic understanding leaves a somewhat descriptive taste to this study, but this reviewer does not think that his detracts from the overall impact of this paper. Many studies will be spawned by this paper, including efforts to understand the molecular and cellular mechanisms by which RA signaling governs the specification of the various utricular sub-zones.

Reviewer #3 (Remarks to the Author):

This paper describes a role for retinoic acid (RA) signaling in the patterning of vestibular sensory epithelia and then shows how a change in patterning can alter specific vestibular behaviors. The vestibular system detects the movement of the head, information that is important for balance, postural control, and gaze stabilization. Linear acceleration is detected by the maculae of the utricle and saccule. Angular acceleration is detected by the cristae of the three semicircular canals. The maculae and crisate are further patterned from the central to peripheral regions. In particular, the central, striolar zones are mostly innervated by irregular vestibular ganglion neuron (VGN) afferents, while the peripheral, extrastriolar zones are innervated by VGN afferents with more regular firing properties. Despite these and many other distinct anatomical differences, including otoconia

composition and hair cell bundle morphology, it is not clear how these regions are patterned during development nor how either region specifically contributes to vestibular function. In this study, the authors provide strong evidence that RA signaling patterns the central/peripheral zones of the vestibular epithelia and go on to show that the striolar zone is critical for VsEP responses, for walking normally along a balance beam, and for stabilizing the head, but not for many other classic vestibular behaviors, such as the vestibulo-ocular reflex or many other vestibulo-spinal reflexes.
This is an important and interesting study for the field, akin to showing both how the fovea of the eye forms and why it is important for vision. The team nicely illustrates the complementary expression of the RA degrading enzyme Cyp26b1 and the synthesizing enzyme Aldh1a3 in the vestibular sensory epithelia. They performed an impressively thorough analysis of the central/peripheral organization of mice lacking each of these genes and found largely opposite results: the striola was essentially lost from Cyp26b1 mutants and expanded in Aldh1a3 mutants (though not all epithelia were affected in this strain). These phenotypes are quite convincing, with clear changes not only in the overall morphology and organization of cells in the central vs peripheral zones, but even in the electrophysiological properties of the innervating afferents. The suite of behavioral tests was similarly extensive and uncovered a selective effect in Cyp26b1 mutants that strongly implicates the striola in the generation of the VsEP, which is the summated response to linear jerks of the head. Throughout, the authors reported extensive statistical analyses to further strengthened the conclusions. Altogether, the findings are new, the experiments were well done and appropriately analyzed, and the interpretations are appropriate. I have only minor criticisms.
1. It is remarkable how completely the striola seems to be lost from the Cyp26b1 null mutants and conditional knock-out animals. What I found hard to understand, though, is why the afferents change. It was not clear to me if this means that RA patterns the "identity" of irregular vs regular VGN neurons or whether they take on different properties based on the broader properties of the region they innervate (could the change in the structure of the otoconia or the hair bundle morphologies contribute, for instance?). A bit more information about whether these changes are secondary or might instead be primary (is cyp26b1 expressed in the VGNs?) would be helpful.
2. Along the same lines, there is a note in the discussion that Cyp26b1 is also expressed in the brain (unsurprisingly), which could of course impact the behavioral studies. The authors should perform in situ's of Cyp26b1 in the vestibular brainstem and cortex and show the data so that readers can think more independently about whether any of the observed effects could originate centrally.
3. In Figure 4, traces from control extrastriolar fibers should be shown for comparison.
4. Although the paper is well written and the data are convincing, the discussion was not especially insightful. In particular, I would have liked to see more discussion of the parallels between the striola and the fovea, the possible role of RA there, and also some more discussion of why certain behaviors depend on the striola. This would make the paper much more generally accessible to non-experts in particular, but would also strengthen the overall conclusions regarding the function of the striola. The introduction could be improved similarly, by providing more information about what is known about RA in particular.
5. Line 91: there is a typo: "are" should be added before "more sensitive".
6. Line 111: RA is not a transcription factor. Its receptor functions as one. This should be corrected.

Reviewer #4 (Remarks to the Author):

Previous work has shown that retinoic acid (RA) is a critical regulator of inner ear development, affecting in particular the antero-posterior patterning of the otic vesicle. In this very elegant and thought-provoking study, the authors uncover a new role for a RA degradation enzyme, cyp26b1, in the formation of the central region of the vestibular sensory epithelia. This region (also called the striola in the vestibular maculae) is anatomically, molecularly and physiologically distinct from the more peripheral region, but its specific contribution to vestibular function was not clear. Based on a series of electrophysiological and behavioural tests of vestibular function in the cyp26b1 cKO, the authors conclude that the central region of the utricle is not required for vestibulo-ocular reflexes, but

is important for performing ‘challenging’ vestibulo-motor activities and for the maintenance of head stability.

The manuscript is very well written and the figures are clear. The presentation of the data (box/dot plots in most cases) is very good, there is a real effort to make the methodology and results accessible to a wide readership. I am not expert with the physiology/electrophysiology/behavioural techniques used in the second part of the manuscript and cannot comment on this aspect of the work, but I was impressed by the extensive battery of tests of vestibular function performed by the authors - some of these leading to very unexpected results, such as the better Rotarod performance of the Cyp26b1 cKO! I have on the other hand more comments and specific questions about the role of cyp26b1 in the differentiation of the vestibular organs:

- The phenotypes of the Aldh1a3 and cyp26b1 deficient mice are very intriguing and raise the possibility that RA has a morphogen-like function in the developing vestibular epithelia, acting in a dose-dependent manner to specify regional cell identities. However the phenotype of the cyp26b1 mice is complex and more experiments would be needed to determine whether RA levels do indeed 'specify' the identity of the central regions of the vestibular epithelia. For example, there is a strong reduction in the number of oncomodulin-positive hair cells in the Cyp26b1-/- mice, but the supporting cell marker for the striolar region (beta-tectorin) is still detected in the right place, although in a reduced domain. This suggests that the specification (identity) of this domain is not entirely compromised but perhaps its normal growth, differentiation, or survival are. The authors themselves conclude that “striolar maturation depends on low levels of RA signaling” (line 263), which is another interpretation of the data… I think it is impossible to distinguish between these possibilities with the present results and some follow-up experiments will be necessary to clarify the exact role of RA in the development of the vestibular sensory epithelia. Whilst this is beyond the remit of this paper, which is really about the contribution of the central region to vestibular function, the wording of the title (“specification” of vestibular striola) and other statements (line 40 “identities”, line 109 “specification”, line 148 “specification…requires”) should be revised to account for these uncertainties regarding the function of RA.

- What is the significance of the reduced expression of oncomodulin in the cKO utricle? Does this reflect a reduction in type I HC numbers, or a reduction in expression of oncomodulin in type I HC (otherwise present in normal numbers)? I could not find any clear mention of an effect (or not) on type I HC numbers and was left confused by this result, which is important for the interpretation of the physiological data. Perhaps some high mag images of sensory epithelia co-labelled with combinations of antibodies for oncomodulin/myo7a on transverse views generated from confocal stacks to highlight HC morphology (is that sufficient to decide on type I versus II?) should be shown to address these points.

- Is the reduction in calbindin expression, which labels afferents of the striolar region, a potential consequence of a reduction in type I HC numbers? Is there a spatial correlation between remaining oncomodulin+ HC (normal type I HC/striolar) and calbindin expression in the cKO utricle?

- Have the authors looked at GATA3 expression, which is (in the chicken at least) a good marker for the striola region? This may be another way to examine the question of specification versus other roles for RA.

- Figure 1a-b-c: consider including a single channel image for oncomodulin. It seems from figure suppl2 that some cells have reduced levels of oncomodulin in the saccule and I wondered whether the same might be the case in the utricle/crista?

- Figure 1f-g-h and j-k-l: change to grey levels given that these are single-channel images?

- Line 204: repetition of “expanded”

Nicolas Daudet

DEPARTMENT OF HEALTH & HUMAN SERVICES

Public Health Service
National Institutes of Health

National Institute on Deafness
and Other Communication Disorders
Bethesda, Maryland 20892
Building: 35A Convent Dr
Room :1D824
(v) (301) 402-4214
(FAX) (301) 402-5475
(E-Mail) wud@nidcd.nih.gov

Sept 10,2019

Dear Editor,

We thank the reviewers for their positive comments and overall enthusiasm for our manuscript. In response to reviewers' comments, we have revised Figures 2 and 4 in the main text as well as revised Suppl Fig. 8 and included 3 additional supplemental figures (Fig. 2, 5, and 6). All the changes pertinent to reviewers' suggestions are highlighted in red. The revised manuscript including Introduction, Results and Discussion, have a total of 5873 words and are within the word limit set by the journal. Our point-by-point responses to the comments raised are listed as follow:

Reviewer #1:

The report by Ono et al. show that formation of the striolar/central zones of the vestibular organs in mice during embryogenesis requires Cytochrme P450. In Cyp26b1 KO mice the authors describe how the distinctive identities of these zones are compromised. This includes a reduction in the numbers of Type ! hair cells within the striolar/central zones, a reduction in the number of calyx-only and complex calyx endings, and loss of size differentiation among otoconia over the strioloar zone. Physiologically, there were no differences in threshold currents between striolar and extra-striolar afferents.

Because of their unique dynamic properties, irregular afferents from the striolar region are believed to be the primary generators of the vestibular short latency evoked potential (VsEP). Consistent with the observed morphological and physiological changes, the VsEP was significantly altered or absent in the Cyp26b1 KO mice. Interestingly, these mice performed better on roto-rod tests and appeared to have relatively normal VOR and OVAR responses.

This paper reports an extensive array of experimental maneuvers focussed on the development of the striolar/central zones in mice and on vestibular behaviors that may be dependent on this specialization. The data are presented in a logical order and clearly described. The behavioral data including the VsEP measurements are relatively standardized and performed by experienced investigators. Although the number of animals used in any one test was small, the data were statistically significant and the differences between control and KO mice were large and evident.

This is a paper describes several notable findings of particular interest to vestibular scientists (e.g., the sources of the VsEP and the contribution of extra-striolar afferents to the VOR and to vestibulo-spinal reflexes) and developmental mechanisms that determine specific sensory specializations. Although the data of individual maneuvers are presented briefly and succinctly, the overall conclusion appears to be well founded and will be of broad interest to the field.

We thank the reviewer for the positive comments on our manuscript.

Reviewer #2:

It is well established that the vestibular maculae are divided into anatomically well-distinguished zones, but their relevance for specific vestibular functions is poorly understood. This study by Ono et al. makes a decisive contribution to fill this gap in knowledge. Based on overall very convincing data, the authors show that the specification of the central/striola part of the utricle depends on RA signaling. By genetically manipulating the developmental specification of this zone, they show that the central/striola zone is essential for detecting transient responses in linear acceleration. The conclusions are supported by data of excellent quality, with the exception of a few points that will be outlined below.

It is well known that RA gradients, established by RA producing sources and metabolizing sinks, are important for setting up certain axis during organ development, and a previous study (Romand et al) demonstrated that Aldh1a3 (a RA source) and Cyp26b1 (a sink) exhibit complementary expression patterns in the cochlea. The findings presented in this paper therefore are not entirely surprising or conceptually new. However, this study's genetic deletion of Cyp26b1 produces a strikingly specific and clean phenotype (loss of striola specification), which allowed the authors to definitively dissect the functional role of the utricular striola. The complete loss of VsEP in a mouse that is normal in most other vestibular functions is a powerful phenotype, and provides a useful basis to dissect out the different vestibular modalities.

What prevents this study from being complete is the lack of any effort to dissect the mechanism by which RA signaling leads to the zonal specification, including the cellular phenotypes in SC and HCs. This reviewer however thinks that this manuscript has sufficient novelty, significance and substance to justify publication in a high-profile journal, without delineating a clear mechanistic understanding, provided the following concerns can be addressed in a satisfactory manner:

- Figure 1: the expression pattern of Aldh1a3, Cyp26b1 and β-tectorin mRNA in the vestibular macula is convincing and consistent with previous reports in the cochlea, and the quality of the in situs is appropriate. It would be better however to show the protein expression pattern. The authors must have tried immunofluorescence. Did the IFs not work? IFs would allow a more convincing confirmation that Aldh1a3 and Cyp26b1 are indeed expressed in SCs.

We agree with the reviewer that Aldh1a3 and Cyp26b1 immunostaining are better for determining cell-type localization. Unfortunately, we have not yet come across an antibody that works for Cyp26b1. We do, however, have success with a commercial antibody against Aldh1a3, and our immunostaining results confirmed the in situ results that Aldh1a3 is mainly expressed in the extrastriolar/peripheral zone of vestibular organs at E15.5. We also show that by P0, Aldh1a3 immunostaining is present in both HCs and SCs of the extrastriolar region, and in some striolar HCs of the utricle. These results were also verified by in situ hybridization. The immunostaining results are now included as Suppl. Fig.2 and described in the Results on P.6.

- When the Aldh1a3 and Cyp26b1 constitutive KO mice are introduced the first time, it would be helpful to mention that these mice die around birth, but are ok for embryonic studies. This also sets up the rationale for the conditional KOs.

Sentence added on P.6.

- Line 156 states that there is no apparent loss of total number of MYO7A positive hair cells, without providing any data. This needs to be quantified,
especially since later in the manuscript, it is reported that the density of hair cells changes in the striolar region of the conditional Cyp26b1 mice (line 232).

Quantification of the total number of Myo7a positive HCs showed a 13% increase in Cyp26b1 mutant utricles and no change in Aldh1a3 mutants. These results have been added to the text (P.7) and an additional graph, Fig.2d.

- Line 194: The authors state: "In Cyp26b1 cKO mice, Ocm and β-tectorin phenotypes are similar to those of the Cyp26b1 knockouts (data not shown)." Data not shown is not acceptable in this case. The authors need to provide data for this.

We have provided the data and quantification in Suppl Fig. 5 of the revised manuscript.

- Starting line 238, the authors investigate morphological phenotypes in hair cells in the Cyp26b1 mutant versus the WT mice:
a) it would be good to learn whether the number or ratio of type I and type II hair cells changes in the mutant mice. This has not been investigated in this study.

Note that we reported an increase in total HC density in the striolar zone of mutant relative to WT, consistent with increased ES identity (ES zone has higher HC density in control animals). In this revision, we further demonstrate the presence of osteopontin, an extrastriolar type I HC marker (McInturff, Burns, Kelley, 2018), in the striola of mutants, providing strong evidence that the type I HCs in the mutant striola have taken on some extrastriolar identity.
In addition, as the reviewer requested, we compared type I and II HC counts and ratios in control and cKO utricles using anti-Sox2 (labels SCs and type II HCs) and anti-spectrin antibodies (labels all HCs). First, similar to other studies, we found no difference between the ratio of type I and II HCs in the striola and extrastriola of controls (see Figure below). We saw no decrease in the number or % of type I HCs in the mutant striolas, rather a slight increase compared to controls. We have not added this figure to the revised paper because (1) type I:II ratio is not a good marker for zone, and (2) we show other evidence that type I HCs are plentiful in the mutant striola.

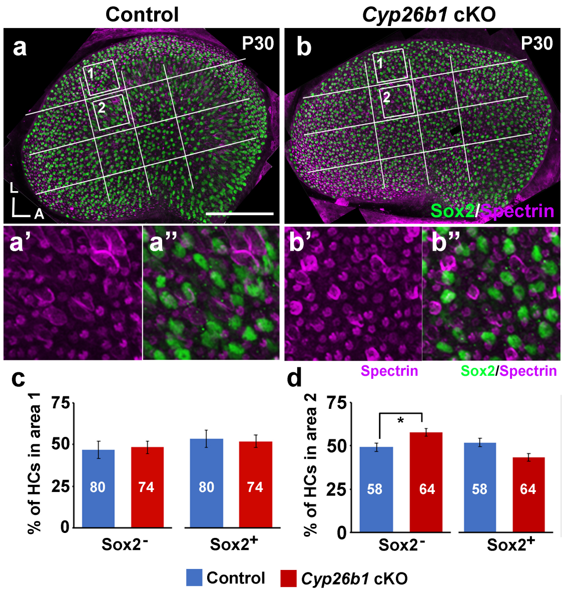

Figure. Quantification of type I and type II HCs in *Cyp26b1* cKO utricles. Immunohistochemistry (a-b") and quantification of Sox2-positive (Sox2^{+}, green) and Sox2-negative (Sox2^{-}) HCs in P20-30 utricles (c,d). Anti-Spectrin antibodies (magenta) labels all HCs. Areas 1 and 2 correspond to the LES and striola, respectively. Sox2^{+} Type II HCs and Sox2^{-} Type I HCs are distributed equally in both extrastriola (**a**, **c**, 53.3 ± 5.1 Sox2^{-} HCs vs. 46.6 ± 5.1 % Sox2^{+} HCs in area 1, n = 3) and striola (**a-a"**, **d**, 50.8 ± 2.2 Sox2^{-} HCs vs. 49.1 ± 2.2 % Sox2^{+} HCs in area 2, n = 3) of control utricles. In contrast, percentages of Sox2^{-} HCs are increased in area 2 (**b-b"**, **d**, 57.6 ± 2.1 % Sox2^{-} HCs vs. 42.3 ± 2.1

$Sox2^-$ HCs, n = 3) but unchanged in the LES (**b**, **c**, 48.2 ± 3.7 % $Sox2^-$ HCs vs. 51.7 ± 3.7 $Sox2^+$ HCs, n = 3, p = 0.807) of *Cyp26b1* cKO utricles. The increase in % of $Sox2^-$ HCs in mutants is not significantly different from controls (asterisk, 2-way ANOVA). Numbers within bars indicate the total number of HCs counted for the area. Scale bar; 200 μm. A, anterior; L, lateral.

b) the immunofluorescence images used to quantify the kinocilium/stereocilia length ratio are of insufficient quality. Please provide better IF images, and use imaging tools such as Imaris to render 3D stacks and determine lengths of stereocilia and kinocilia in 3D space. Or better, conduct analysis on SEMs.
c) in addition to the relative ratio, the absolute length is here of interest. Especially since a failed coupling of the kinocilia/stereocilia with the gelatinous zone/otoconia might cause the functional defects.
Improving the images of the hair bundles would require different methods and a significant extension of time. In response to the reviewer's comment, we have increased the sample numbers and quantified the kinocilium/stereocilia length ratio using the Imaris software. Those data are now included in Supplemental Fig. 8.
With respect to disruption of coupling: The collective evidence suggests that in the striolar zones of Cyp26b1 mutants, both the accessory structures (otolithic gel and otoconia) and the hair bundles have the morphology typical of the ES zone in both controls and mutants. Thus, bundle-gel coupling in the mutant striola is likely to be as found in control ES zones, where relatively tall kinocilia project into the otolithic gel, and therefore effective at transmitting head motion stimuli. The loss of the kind of mechanical coupling typical of control striolas (with a more porous gel, different otoconia, and shorter kinocilia) is indeed likely to contribute importantly to the vestibular phenotype.

Discussion:
- it seems the RALDH3 mouse phenotype differs from a previous study. Any idea why that would be the case?
The previous published results did not specifically report striola and central zone phenotype in the Raldh3 mutants and results from the two studies are not in conflict. Furthermore, the VOR and OVAR deficits reported in the previous study are consistent with our hypothesis that the extrastriola and peripheral zones are more important for VOR functions.

Reviewer #3:

This paper describes a role for retinoic acid (RA) signaling in the patterning of vestibular sensory epithelia and then shows how a change in patterning can alter specific vestibular behaviors. The vestibular system detects the movement of the head, information that is important for balance, postural control, and gaze stabilization. Linear acceleration is detected by the maculae of the utricle and saccule. Angular acceleration is detected by the cristae of the three semicircular canals. The maculae and crisate are further patterned from the central to peripheral regions. In particular, the central, striolar zones are mostly innervated by irregular vestibular ganglion neuron (VGN) afferents, while the peripheral, extrastriolar zones are innervated by VGN afferents with more

regular firing properties. Despite these and many other distinct anatomical differences, including otoconia composition and hair cell bundle morphology, it is not clear how these regions are patterned during
development nor how either region specifically contributes to vestibular function. In this study, the authors provide strong evidence that RA signaling patterns the central/peripheral zones of the vestibular epithelia and go on to show that the striolar zone is critical for VsEP responses, for walking normally along a balance beam, and for stabilizing the head, but not for many other classic vestibular behaviors, such as the vestibulo-ocular reflex or many other vestibulo-spinal reflexes. This is an important and interesting study for the field, akin to showing both how the fovea of the eye forms and why it is important for vision. The team nicely illustrates the complementary expression of the RA degrading enzyme Cyp26b1 and the synthesizing enzyme Aldh1a3 in the vestibular sensory epithelia. They performed an impressively thorough analysis of the central/peripheral organization of mice lacking each of these genes and found largely opposite results: the striola was essentially lost from Cyp26b1 mutants and expanded in Aldh1a3 mutants (though not all epithelia were affected in this strain). These phenotypes are quite convincing, with clear changes not only in the overall morphology and organization of cells in the central vs peripheral zones, but even in the electrophysiological properties of the innervating afferents. The suite of behavioral tests was similarly extensive and uncovered a selective effect in Cyp26b1 mutants that strongly implicates the striola in
the generation of the VsEP, which is the summated response to linear jerks of the head. Throughout, the authors reported extensive statistical analyses to further strengthened the conclusions. Altogether, the findings are new, the experiments were well done and appropriately analyzed, and the interpretations are appropriate. I have only minor criticisms.

1. It is remarkable how completely the striola seems to be lost from the Cyp26b1 null mutants and conditional knock-out animals. What I found hard to understand, though, is why the afferents change. It was not clear to me if this means that RA patterns the "identity" of irregular vs regular VGN neurons or whether they take on different properties based on the broader properties of the region they innervate (could the change in the structure of the otoconia or the hair bundle morphologies contribute, for instance?). A bit more information about whether these changes are secondary or might instead be primary (is cyp26b1 expressed in the VGNs?) would be helpful.

We totally agree with the reviewer that while the changes in the afferents of Cyp26b1 mutants are clear, the mechanisms that lead to these changes remain elusive. We did not detect Cyp26b1 expression in the developing vestibular neurons by in situ hybridization tested at E13.5 and P0. Therefore, we propose that the changes in afferents are caused indirectly by changes in the supporting cells that drive multiple, coordinated changes at all levels of the organization of the striolar zone, from the otoconia to the afferent terminals. Note that in vivo, striolar afferents are quite different from extrastriolar afferents in terms of spontaneous and stimulus-evoked firing patterns. Removal of all the distinctive features of the striolar input to the afferents would be expected to affect afferent properties – so that it would have been surprising to see no effect on vestibular afferents. The intriguing aspect of these data is that the mutation removes afferent properties that are autonomous to the afferents themselves, including expression of ion channels and calcium-binding proteins. Alternative explanations are possible and we will continue to design experiments to address this question. (See P. 20).

2. Along the same lines, there is a note in the discussion that Cyp26b1 is also expressed in the brain (unsurprisingly), which could of course impact the behavioral studies. The authors should perform in situ's of Cyp26b1 in the vestibular brainstem and cortex and show the data so that readers can think more independently about whether any of the observed effects could originate centrally.
A previous study has shown that Cyp26b1 is expressed in the cerebellum and brainstem during late embryogenesis (Zhang et al., J. Neurosci, 23:7610, 2003). Although Cyp26b1 does not appear to be expressed in the vestibular nuclei, the complexity of the vestibular circuitry in the brainstem and other regions of the brain will not allow us to rule out the possibility of Cyp26b1 loss in the brain not contributing to the behavioral results observed in Cyp26b1 cKO mutants. Thus, we are in the process of generating another mutant line more specific for testing the significance of striola and central zones in mediating challenging vestibular functions.

3. In Figure 4, traces from control extrastriolar fibers should be shown for comparison.
We have added a representative trace from control extrastriolar afferents to Fig4.

4. Although the paper is well written and the data are convincing, the discussion was not especially insightful. In particular, I would have liked to see more discussion of the parallels between the striola and the fovea, the possible role of RA there, and also some more discussion of why certain behaviors depend on the striola. This would make the paper much more generally accessible to non-experts in particular, but would also strengthen the overall conclusions regarding the function of the striola.
The introduction could be improved similarly, by providing more information about what is known about RA in particular.
Due to the word limitation set by the journal, we can only draw parallels between the striola and the fovea concisely (see P.25). We can only refer readers to comprehensive reviews on the role of RA during development (see P.4). We also described briefly the possible function of striolar/central zones (see P.3 for Introd and P.24 for Discussion).

5. Line 91: there is a typo: "are" should be added before "more sensitive".
Corrected

6. Line 111: RA is not a transcription factor. Its receptor functions as one. This should be corrected.
Corrected

Reviewer #4 :

Previous work has shown that retinoic acid (RA) is a critical regulator of inner ear development, affecting in particular the antero-posterior patterning of the otic vesicle. In this very elegant and thought-provoking study, the authors uncover a new role for a RA degradation enzyme, cyp26b1, in the formation of the central region of the vestibular sensory epithelia. This region (also called the striola in the vestibular maculae) is anatomically, molecularly and physiologically distinct from the more peripheral region, but its specific contribution to vestibular function was not clear. Based on a series of electrophysiological and behavioural tests of vestibular function in the cyp26b1 cKO, the authors conclude that the central region of the utricle is not required for vestibulo-ocular reflexes,

but is important for performing 'challenging' vestibulo-motor activities and for the maintenance of head stability.

The manuscript is very well written and the figures are clear. The presentation of the data (box/dot plots in most cases) is very good, there is a real effort to make the methodology and results accessible to a wide readership. I am not expert with the physiology/electrophysiology/behavioural techniques used in the second part of the manuscript and cannot comment on this aspect of the work, but I was impressed by the extensive battery of tests of vestibular function performed by the authors - some of these leading to very unexpected results, such as the better Rotarod performance of the Cyp26b1 cKO! I have on the other hand more comments and specific questions about the role of cyp26b1 in the differentiation of the vestibular organs:

-The phenotypes of the Aldh1a3 and cyp26b1 deficient mice are very intriguing and raise the possibility that RA has a morphogen-like function in the developing vestibular epithelia, acting in a dose-dependent manner to specify regional cell identities. However the phenotype of the cyp26b1 mice is complex and more experiments would be needed to determine whether RA levels do indeed 'specify' the identity of the central regions of the vestibular epithelia. For example, there is a strong reduction in the number of oncomodulin-positive hair cells in the Cyp26b1-/- mice, but the supporting cell marker for the striolar region (beta-tectorin) is still detected in the right place, although in a reduced domain. This suggests that the specification (identity) of this domain is not entirely compromised but perhaps its normal growth, differentiation, or survival are. The authors themselves conclude that "striolar maturation depends on low levels of RA signaling" (line 263), which is
another interpretation of the data… I think it is impossible to distinguish between these possibilities with the present results and some follow-up experiments will be necessary to clarify the exact role of RA in the development of the vestibular sensory epithelia. Whilst this is beyond the remit of this paper, which is really about the contribution of the central region to vestibular function, the wording of the title ("specification" of vestibular striola) and other statements (line 40 "identities", line 109 "specification", line 148 "specification…requires") should be revised to account for these uncertainties regarding the function of RA.

We thank the reviewer for the insightful comment and we agree that the word "specification" may be too strong and we have revised the manuscript accordingly and changed the word from "specification" to "formation", including the title.

- What is the significance of the reduced expression of oncomodulin in the cKO utricle? Does this reflect a reduction in type I HC numbers, or a reduction in expression of oncomodulin in type I HC (otherwise present in normal numbers)? I could not find any clear mention of an effect (or not) on type I HC numbers and was left confused by this result, which is important for the interpretation of the physiological data.
Perhaps some high mag images of sensory epithelia co-labelled with combinations of antibodies for oncomodulin/myo7a on transverse views generated from confocal stacks to highlight HC morphology (is that sufficient to decide on type I versus II?) should be shown to address these points.

The reviewer raised an excellent question of whether type I HCs are still present in the "striolar/central" region of cKO mutants. To address this question, we conducted additional analyses using anti-osteopontin antibody, which labels extrastriolar type I HCs. Our results show that osteopontin staining is upregulated in the center region of the utricle compared to controls (Supplemental Fig.6).

These results suggest that type I HCs are still present in the striola region of mutants but they have taken on some extrastriolar type I identity.

- Is the reduction in calbindin expression, which labels afferents of the striolar region, a potential consequence of a reduction in type I HC numbers?

No, because as we now show there is not a reduction in the number of striolar type I HCs in mutants. See figure and discussion above in response to related comments from Reviewer 2.

Is there a spatial correlation between remaining oncomodulin+ HC (normal type I HC/striolar) and calbindin expression in the cKO utricle?

Whether there is a spatial correlation between remaining Ocm+ HCs and calbindin expression in the neurons is a good question. However, it won't be an easy result to obtain or interpret. If we find the residual Ocm+ HCs spatially correlated with the calbindin+ staining in the mutants, we will not be able to conclude whether the spatial correlation of calbindin staining with Ocm is directly due to the presence of Ocm+ HCs or indirectly due to residual Cyp26b1 activity. There is simply not enough known about whether neuronal development is dependent on HCs in normal or mutant conditions. It is also a difficult result to obtain because most of our specimens do not show much Ocm+ or calbindin+ staining. We will need to collect a large number of mutants before we can be confident with our analyses. We have prepared some samples for this purpose but failed to obtain sufficient Ocm+ or calbindin+ staining to make a conclusion and we decided not to further delay the resubmission of this manuscript. This question will be addressed in a separate study.

- Have the authors looked at GATA3 expression, which is (in the chicken at least) a good marker for the striola region? This may be another way to examine the question of specification versus other roles for RA.

Gata3 is expressed in the mouse striola similar to the chicken. This expression also appears to be downregulated in the Cyp26b1$^{-/-}$ mutants. We are still in the process of analyzing the relationships between Cyp26b1 and Gata3 for our mechanistic study of RA in the utricle and the results will be included in a separate manuscript.

- Figure 1a-b-c: consider including a single channel image for oncomodulin. It seems from figure suppl2 that some cells have reduced levels of oncomodulin in the saccule and I wondered whether the same might be the case in the utricle/crista?

Due to space constraints in the main text, single-channel images are provided in Supplemental Fig. 5. Yes, reduced levels of oncomodulin were observed in the utricle and cristae similar to the saccule.

- Figure 1f-g-h and j-k-l: change to grey levels given that these are single-channel images?s

We did not change the image to grey scales in order to stay true to the in situ hybridization results.

- Line 204: repetition of “expanded”
Corrected

I hope you will find our revised manuscript acceptable for publication and we look forward to hearing from you.

Sincerely,

Doris Wu, Ph.D.
Principal Investigator

REVIEWERS' COMMENTS:

Reviewer #2 (Remarks to the Author):

This manuscript by Ono et al. was quite excellent to begin with, and the few holes in the initial submission were successfully fixed in the revised manuscript: the authors made a good faith effort to detect Aldh13 and Cyp26b1 on the protein level, and succeeded in doing that for the former. The results are convincing and complement the in situs. The other minor points have also been addressed.

While I didn't bring up this issue in my review, I agree that the change of the title is appropriate.

I have no further revision requests.

This is an important work for not only the inner ear field, but also for the wider neuroscience community, by providing the basis for the formation of the anatomical and cellular correlate of an important vestibular submodality.

Reviewer #3 (Remarks to the Author):

This was already a very nice paper and the revision is even better. I do not have any major criticisms.

I did find a couple of typos, though:

Line 144: "the" is missing from "as sensory epithelium matures"
Line 190: "postnal" should be "postnatal"
Line 508: "estbiular" should be "vestibular"
Lines 151-155: It would be good to make it clear what age you are examining, not just the fact that the animals die at birth.

Reviewer #4 (Remarks to the Author):

The authors have addressed all of my main comments appropriately and have provided very interesting supplementary data (osteopontin immunostaining/quantification) that supports their original hypothesis regarding the change in type I HC character in the Cyp26cKO.I am satisfied with the revised version of the manuscript.

Dear Editor,

We thank you and the reviewers for their positive comments and overall enthusiasm for our manuscript. Our point-by-point responses to reviewers' comments as follow:

REVIEWERS' COMMENTS:

Reviewer #2 (Remarks to the Author):

This manuscript by Ono et al. was quite excellent to begin with, and the few holes in the initial submission were successfully fixed in the revised manuscript: the authors made a good faith effort to detect Aldh13 and Cyp26b1 on the protein level, and succeeded in doing that for the former. The results are convincing and complement the in situs. The other minor points have also been addressed.
While I didn't bring up this issue in my review, I agree that the change of the title is appropriate.
I have no further revision requests.
This is an important work for not only the inner ear field, but also for the wider neuroscience community, by providing the basis for the formation of the anatomical and cellular correlate of an important vestibular submodality.
We appreciate the reviewer's positive comments on our manuscript.

Reviewer #3 (Remarks to the Author):

This was already a very nice paper and the revision is even better. I do not have any major criticisms.

I did find a couple of typos, though:

Line 144: "the" is missing from "as sensory epithelium matures"
Line 190: "postnal" should be "postnatal"
Line 508: "estbiular" should be "vestibular"
All corrected
Lines 151-155: It would be good to make it clear what age you are examining, not just the fact that the animals die at birth.
Age information has been added.

Reviewer #4 (Remarks to the Author):

The authors have addressed all of my main comments appropriately and have provided very interesting supplementary data (osteopontin immunostaining/quantification) that supports their original hypothesis regarding the change in type I HC character in the Cyp26cKO.I am satisfied with the revised version of the manuscript.

We thank the reviewer's positive comments on our manuscript.

I hope you will find our revised manuscript acceptable for publication and we look forward to hearing from you.

Sincerely,

Doris Wu, Ph.D.
Principal Investigator
